# Reevaluating pragmatic reasoning in language games

**DOI:** 10.1371/journal.pone.0248388

**Published:** 2021-03-17

**Authors:** Les Sikos, Noortje J. Venhuizen, Heiner Drenhaus, Matthew W. Crocker

**Affiliations:** Department of Language Science and Technology, Saarland University, Saarbrücken, Germany; Ball State University, UNITED STATES

## Abstract

The results of a highly influential study that tested the predictions of the Rational Speech Act (RSA) model suggest that (a) listeners use pragmatic reasoning in one-shot web-based referential communication games despite the artificial, highly constrained, and minimally interactive nature of the task, and (b) that RSA accurately captures this behavior. In this work, we reevaluate the contribution of the pragmatic reasoning formalized by RSA in explaining listener behavior by comparing RSA to a baseline literal listener model that is only driven by literal word meaning and the prior probability of referring to an object. Across three experiments we observe only modest evidence of pragmatic behavior in one-shot web-based language games, and only under very limited circumstances. We find that although RSA provides a strong fit to listener responses, it does not perform better than the baseline literal listener model. Our results suggest that while participants playing the role of the Speaker are informative in these one-shot web-based reference games, participants playing the role of the Listener only rarely take this Speaker behavior into account to reason about the intended referent. In addition, we show that RSA’s fit is primarily due to a combination of non-pragmatic factors, perhaps the most surprising of which is that in the majority of conditions that are amenable to pragmatic reasoning, RSA (accurately) predicts that listeners will behave non-pragmatically. This leads us to conclude that RSA’s strong overall correlation with human behavior in one-shot web-based language games does not reflect listener’s pragmatic reasoning about informative speakers.

## 1 Introduction

Understanding language successfully often requires listeners to go beyond the literal meaning of an utterance. If, for example, your dinner guest asks you to “Pass the fish” in a situation where there are two fish dishes on the table, you will likely infer that the speaker is referring to the dish that is closer to you. After all, why would they ask you to pass the dish that is already near them? A large body of theoretical work has attempted to describe such instances of pragmatic reasoning and other related phenomena (e.g., [1–10]). Most prominently, Grice [4] presented *conversational implicature theory*, an initial framework for understanding how speakers and listeners flexibly use language to achieve their social goals. In line with this account, cooperative speakers should choose their utterances to be optimally informative. Pragmatic listener behavior can then be described in terms of a set of *conversational maxims* that allow an optimal listener to work backwards from the specific utterance in order to infer the speaker’s intended communicative goal.

**Fig 1:**
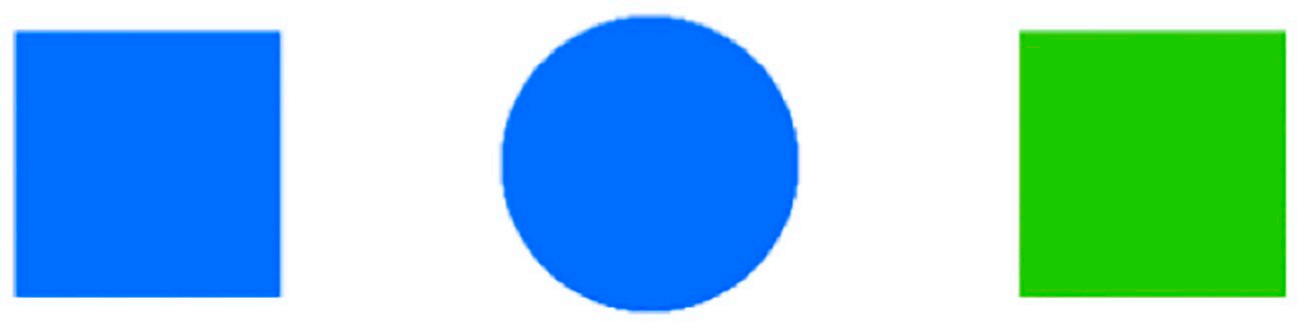
Example context type from F&G [20].

Much recent work has focused on constructing formal models of pragmatic reasoning (e.g., [11–24]; see [25], for a review) Critically, the predictions of many of these models have been extensively tested against empirical data on human reasoning behavior. A highly influential paper in this regard is the *Science* article by Frank & Goodman [20] (henceforth, F&G), which introduces the *Rational Speech Act* (RSA) model of human pragmatic reasoning and evaluates its performance against behavioral data in a referential communication game [26]. Referential communication games attempt to capture in a controlled manner the kind pragmatic inferencing people naturally seem to adopt in situations like the “pass the fish” example described above. Highly simplified laboratory versions of this game have been used to investigate the production and comprehension of referential expressions in a wide variety of studies (e.g., [20, 23, 27–38]). In a referential communication game, participants are presented with a visual context that contains a set of objects whose features (e.g., color, shape, pattern) critically do or do not overlap. An example visual context from F&G is shown in Fig 1. Participants assigned to the *Speaker* role have to describe one of the objects, typically by using a one-word message (e.g., “blue” or “circle”), while participants assigned to the *Listener* role attempt to identify the intended referent after observing the given word. In addition, these studies generally incorporate a *Salience* task, in which participants are asked to identify the object that a speaker is most likely to talk about, in order to estimate any prior preferences due to the visual context alone.

When applied to such a game, the RSA model uses Bayesian inference to probabilistically estimate which referent listeners will select from a visual context, based on the given (one-word) utterance. A *pragmatic* listener—as formalized by the RSA model—reasons about the likelihood that an informative speaker would utter a particular word to refer to a referent. For instance, given the word “blue” in the context shown in Fig 1, the pragmatic listener can infer that the intended referent is the *blue square*, because for the alternative referent (the *blue circle*) there exists a more informative expression (“circle”). In contrast, a non-pragmatic ‘literal’ listener is predicted to simply guess between the referents that match the literal interpretation of the given word (i.e., the *blue square* and the *blue circle*). Results from a one-shot web-based version of the referential communication game showed that predictions from the RSA model were almost perfectly correlated with human judgments (*r* = 0.99). Indeed, ensuing work has shown that the RSA framework can be successfully applied to capture listener behavior in a wide variety of domains, including manner implicatures [39], figurative meaning [40], plural predication [41], and over-informative referring expressions [42].

Importantly, however, the original results from the F&G study arguably present the most surprising—and therefore perhaps the most compelling—evidence for the RSA model, as this study employed a particularly artificial, constrained, and minimally interactive version of the referential communication game that does not appear to provide listeners with much motivation to engage in pragmatic inferencing. In particular, F&G used a *one-shot* paradigm, which means that participants were tested on a single trial, and did not get any time to familiarize themselves with the task. Nevertheless, F&G report a high correlation to the RSA predictions, which has been taken to suggest that (a) listeners indeed use pragmatic reasoning in this version of the task, and (b) RSA accurately captures this behavior. A closer look at their results suggests, however, that the evidence for pragmatic reasoning as formalized by RSA may not be as strong as previously assumed. That is, among the full set of visual contexts tested by F&G, only a small subset of trials (approximately 17%) require the type of pragmatic reasoning illustrated in Fig 1 (we will refer to such trials as *pragmatically solvable*). Moreover, it is not clear from the results presented by F&G whether listeners actually reasoned about an informative speaker in these pragmatically solvable conditions. In fact, follow-up studies [32, 43] that focused on the pragmatically solvable conditions found little evidence of pragmatic reasoning in these contexts. To account for these results, both [32] and [43] proposed various modifications to the basic RSA model (e.g., adding parameters for speaker/listener degree of rationality, depth of recursion, etc.), but neither study questioned whether the underlying pragmatic machinery of the RSA model was necessary for explaining human behavior in this task, nor was the performance of RSA evaluated relative to a plausible baseline model that does not assume that listeners reason about informative speakers.

In this paper we present a set of experiments that aim to investigate the extent to which the pragmatic reasoning assumed by the RSA model is required to explain human judgments in one-shot web-based communication games. To address this question, we systematically explore a broader range of visual context types in order to assess which contexts elicit pragmatic behavior in this task, and under what circumstances. In addition, we evaluate the performance of RSA relative to a baseline literal listener model that does not assume that listeners reason about informative speakers. We show that the basic RSA model does not provide a better fit to the overall behavioral data set than the baseline literal listener model, which is essentially driven by the prior probability of referring to an object (measured empirically via the Salience task). In fact, we only find modest evidence of pragmatic behavior in this task and only under very limited circumstances, which cannot be fully explained by the basic RSA model. These results suggest that listeners do not reason as pragmatically as previously assumed in these referential communication games.

In what follows, Section 2 provides a brief overview of the one-shot web-based referential communication game and a detailed description of the basic RSA model from F&G. Section 3 introduces the current study and the literal listener model that we use as a baseline. We then present the three experiments that investigate (i) pragmatic reasoning in a broader range of context types (Section 4), (ii) the role of the prior in pragmatically solvable contexts (Section 5), and (iii) the effect of increased listener engagement on pragmatic reasoning in referential communication games (Section 6). Finally, we discuss our overall results and the implications for formal models of pragmatic reasoning in Section 7.

## 2 Pragmatic reasoning in referential communication games

The RSA model introduced by F&G formalizes pragmatic reasoning behavior in referential communication games. In order to better understand the results presented by F&G, here we describe in detail the one-shot version of referential communication game paradigm, the RSA model itself, and the experimental results reported by F&G.

### 2.1 One-shot web-based referential communication games

In the ‘one-shot’ version of the referential communication game each participant only sees a single trial. In order to collect enough data for statistical analysis, online crowdsourcing tools (e.g., Amazon’s Mechanical Turk) are often used to recruit a large number of participants. A major advantage of this between-subjects approach for referential communication games is that participant responses are not influenced by previous trials, thereby avoiding linguistic convergence effects (e.g., [44]) and other forms of cooperative behavior that can develop over the course of multiple turns [45, 46]. A major disadvantage of the one-shot paradigm, however, is that social interaction is severely limited. As Frank and colleagues acknowledge, “[the one-shot paradigm] eliminates much (but hopefully not all) of the contextual details that create pragmatic inferences in the first place” ([43], p. 37). Indeed, the results of F&G are arguably so compelling because they find evidence of pragmatic reasoning despite the highly constrained and minimally interactive nature of the task.

### 2.2 Rational Speech Act model

F&G introduced RSA as a quantitative model of pragmatic reasoning that can be applied to referential communication games. RSA assumes that a listener can use Bayesian inference to recover the speaker’s intended referent *r*_*S*_ in context *C*, given that the speaker uttered word *w*, as formalized in Eq 1:

$$\begin{array}{c} P\left(r_{S}|w,C\right)=\frac{P\left(w|r_{S},C\right)P\left(r_{S}\right)}{\sum_{r^{\prime }\in C}P\left(w|r^{\prime },C\right)P\left(r^{\prime }\right)} \end{array}$$ (1)

RSA thus models a rational listener who calculates the posterior probability for choosing referent *r*_*S*_, given word *w* and (visual) context *C*, based on two terms: a *speaker model P*(*w*| *r*_*S*_, *C*), which determines the likelihood that a speaker uses word *w* to refer to object *r*_*S*_ in context *C*, and the *prior* probability *P*(*r*_*S*_) of referring to the referent, which is typically operationalised as its contextual salience. A normalizing term ensures that the posterior probabilities of all referents in the context (*r*′ ∈ *C*) form a proper probability distribution. Note that this is in principle a recursive model: Listeners are modeled as reasoning about the decision-making of speakers (i.e. the linguistic costs and benefits of choosing certain expressions), and speakers reason about listeners.

A rational speaker can be modeled according to Bayesian decision theory by defining the probability of words proportional to their expected utility:

$$\begin{array}{c} P\left(w|r_{S},C\right)\propto e^{\alpha U(w;r,C)} \end{array}$$ (2)

where the utility *U*(*w*; *r*, *C*) is defined as the informativeness of word *w* with respect to the speaker’s intended referent (subtracted by its cost), and the *α* parameter determines the degree of speaker rationality (higher *α* means greater maximization of utility). We follow F&G in assuming that word cost is constant in our design, and in quantifying informativeness in terms of word specificity: speakers are more likely to use word *w* to the extent that it reduces referential uncertainty. Based on an *α* parameter set to *α* = 1 (thereby modeling a ‘level-1’ Gricean speaker; see [23]), then, the speaker model is formalized by the following equation (see [20], Supplementary Materials, for a full derivation):

$$\begin{array}{c} P\left(w|r_{S},C\right)=\frac{{\left|w\right|}^{-1}}{\sum_{w^{\prime }\in W}{\left|w^{\prime }\right|}^{-1}} \end{array}$$ (3)

where |*w*| indicates the number of objects in *C* that word *w* can refer to, and *W* is defined as the set of words that can be used to refer to referent *r*_*S*_. Note that this equation implicitly assumes that word *w* can be used to refer to referent *r*_*S*_; if not, *P*(*w*| *r*_*S*_, *C*) is defined to be zero.

By incorporating the speaker model, which defines a probability distribution over words, into RSA’s listener model (Eq 1), RSA yields a predicted probability distribution over the set of referents that are available in visual context *C*. To test the predictions of the RSA model, F&G empirically measured each of the model terms (the *speaker model*, the *prior*, and the *listener model*) using offline human judgments in a one-shot web-based referential communication game.

### 2.3 A closer look at F&G

Using Mechanical Turk, F&G collected separate judgments in a *Speaker* task, a *Salience* task, and a *Listener* task. The Speaker task tested the likelihood *P*(*w*| *r*_*S*_, *C*), as formalized in Eq 3, by asking participants to bet on the word they would use to describe a particular object in a given visual scene. The Salience task estimated the prior probability *P*(*r*_*S*_) that an object will be referred to in a specific visual context by asking participants to bet on the object they think the speaker is referring to when they observe an unknown word. Finally, the Listener task tested the posterior probability *P*(*r*_*S*_| *w*, *C*) resulting from the RSA model (Eq 1) by asking participants to bet on the object they think the speaker is referring to given a single word (e.g. “blue”).

The results revealed a strong correlation between the model’s predictions for informative speakers (Eq 3) and the empirical results from the Speaker task (*r* = 0.98, *p* < .001). As noted above, the model’s posterior predictions for pragmatic listeners (Eq 1) were almost perfectly correlated with the results from the Listener task (*r* = 0.99, *p* < .0001). This correlation remained extremely strong (*r* = 0.87, *p* < .0001) even when removing model predictions of 100% (i.e. when the given word uniquely identified a referent) and 0% (i.e. when the given word excluded a referent). This led the authors to conclude that their model “capture[s] some of the richness of human pragmatic inference in context” ([20], p. 998). Critically, this implies that listeners do in fact use pragmatic reasoning, even in such highly-constrained reference resolution tasks. However, as no baseline was provided, it is unclear whether RSA’s performance is better than what might be expected for a baseline literal listener model that does not incorporate a speaker model (we return to this point shortly).

**Fig 2:**
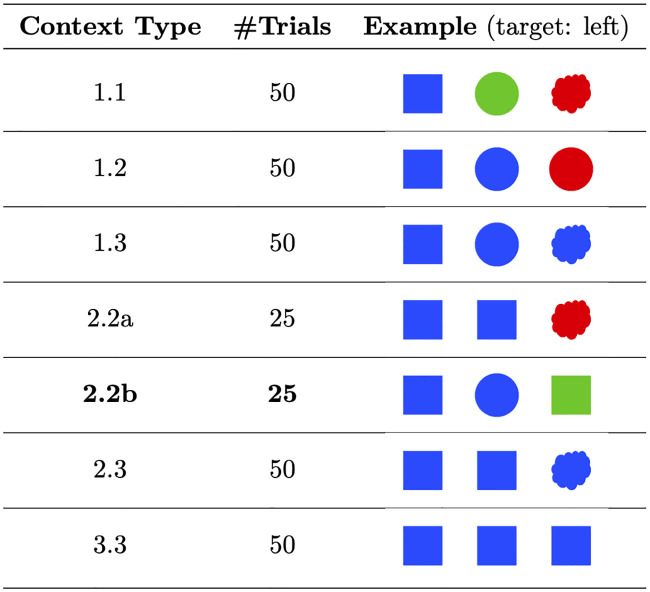
Examples of the visual context types from F&G. The position of the speaker’s target was randomized, but appears on the left in the examples. Context Type indicates the number of objects that share the target’s features (here: #shape.#color). 2.2b is the critical *pragmatically solvable* context type (also shown in Fig 1). See text for details.

Given that the correlation reported by F&G derives from a variety of different visual context types, it is interesting to investigate which contextual features drive this correlation. A closer inspection of the materials used in the study reveals that approximately three-quarters of the items did not benefit from the kind of reasoning RSA is designed to capture. Fig 2 shows an example visual scene from each context type used by F&G. Scenes consisted of a three object display in which different features of the objects were manipulated: color (red/ blue/ green), shape (circle/ square/ cloud), and texture (solid/ polka-dot/ striped). In each scene, two feature dimensions were chosen to vary while the third was held constant (in the examples in Fig 2 texture is held constant). The critical manipulation was the distribution of the target’s features across the objects: F&G systematically varied whether one, two, or three of the objects in the scene had the target’s features. For instance, in context type 1.1 there is a single object with the target’s first feature (shape, in the example shown in Fig 2) and a single object with the target’s second feature (color). This manipulation resulted in six numerical context types, for each of which 50 random trials were generated. In the case of context type 2.2, F&G observed that the number of objects over which the features were distributed (2 objects for context type 2.2a and three objects for 2.2b) affected their model predictions, and therefore 2.2a and 2.2b were separated in the analysis, resulting in only 25 trials for the critical pragmatically solvable context type 2.2b. All analyses were collapsed across features, which resulted in a lack of sensitivity to any potential differences in preference across color versus shape versus texture.

The context types illustrated in Fig 2 show that the kind of reasoning required in the Listener task (identifying the speaker’s intended referent) ranged from trivial to complex. Here we outline how a pragmatic listener (as implemented by RSA) would reason in these context types. In the simplest conditions (e.g., context type 1.1), the literal meaning of the given word (e.g., “blue”) uniquely identifies a single object, thus no pragmatic reasoning is required to resolve the referent. This was the case for approximately one-third of the items. In about 40% of the items, no amount of pragmatic reasoning can overcome the referential ambiguity in the visual scene (e.g., context type 3.3). Therefore, only the remaining one-quarter of items are potentially amenable to pragmatic reasoning and can therefore be considered *pragmatic* contexts; that is, in order to infer the speaker’s intended referent, RSA’s pragmatic listener reasons about the likelihood that the speaker uses the given word to refer to a particular object. Two-thirds of these pragmatic contexts (approximately 17% of the total items) are *pragmatically solvable* (e.g., context type 2.2b), which means that RSA’s pragmatic listener will show a preference for the object which a Gricean account predicts to be the target. For instance, upon observing “blue” in the example for context type 2.2b, RSA predicts that the speaker intends to refer to the *blue square* (the pragmatic referent) because there exists a more informative expression (“circle”) for the *blue circle* (the color competitor). It is important to note that in the remaining one-third of pragmatic contexts (approximately 8% of the total items), the speaker is not fully informative. Nevertheless, RSA predicts that a pragmatic listener will have an equal preference for two of the objects over the third—which reduces but does not completely eliminate referential ambiguity. For example, given the color word “blue” in the example for context type 2.3, a pragmatic listener, as modeled by RSA, infers that the speaker intended to refer to one of the two squares, because the alternative word for the remaining object (*blue cloud*) would be “cloud”, which is more informative than the alternative word for the either of the squares (“square”). Thus, we refer to such contexts as *pragmatically reducible*.

Since F&G do not report behavioral results broken down by context type, we do not know whether listeners responded pragmatically on the critical pragmatically solvable context types. Inspection of Fig 2C in F&G suggests that when listeners observed a color word in the critical pragmatically solvable context type (2.2b), their responses were distributed equally across the pragmatic referent and the color competitor (95% confidence intervals overlap). If anything, there appears to be a numerical preference for the color competitor. It is not clear from Fig 2C how listeners responded in the other half of pragmatically solvable trials, wherein listeners observed a shape word. However, results from the replication study by [32], which only used pragmatically solvable context types, sheds some light on this issue. Table 1 shows that when listeners observed a color word (e.g. “green”) they clearly preferred the pragmatic referent (*green circle*) over the color competitor (*green square*). Salience judgments cannot explain this result because participants deemed the pragmatic referent to be the least salient object. Consequently, this can be taken as fairly strong support for the idea that listeners used pragmatic reasoning to infer the intended referent. Conversely, however, the opposite pattern obtained when listeners observed a shape word (“circle”). In this case, listeners strongly preferred the highly salient shape competitor (*blue circle*). These results for pragmatically solvable trials are therefore mixed: when given a color word, listeners do indeed appear to respond pragmatically (i.e. consistent with RSA’s predictions), but when given a shape word, their responses seem to be driven by salience.

**Table 1:** Behavioral results and an example visual context from [32]. Columns represent choice options: pragmatic referent, color competitor, and shape competitor. Rows present the distribution of responses in the *Salience* task (top row) and *Listener* task when given a color or shape word (bottom rows). Table adapted from [32].

Task	N	pragmatic ref.	color comp.	shape comp.
		*green circle*	*green square*	*blue circle*
Salience	240	0.12	0.30	0.58
Listener—“green”	180	0.64	0.36	0.00
Listener—“circle”	180	0.35	0.00	0.65

In summary, a closer look at the experiment presented by F&G reveals that their reported correlation to human behavioral data may not be reflective of human pragmatic inference in context. It is possible that this strong correlation between the RSA predictions and observed listener judgments was driven primarily by the simpler contexts—constituting the majority of the experimental items—that do not benefit from pragmatic reasoning. Furthermore, it is unclear whether listeners actually responded pragmatically in the contexts that are theoretically amenable to pragmatic reasoning, as illustrated by the mixed results obtained by [32]. One of the critical factors that seems to play a central role in driving the results is the prior probability that an object would be referred to, as measured by the Salience task. Since the prior by itself seems to be an important predictor for listener behavior (at least for a part of the data reported by [32]), it is important to evaluate the extent to which the correlation between RSA’s predictions and the human data is driven by the prior alone. Although F&G found no correlation between the judgments in the Listener and Salience tasks, this might be explained by the observation that in most context types the word that a listener observes excludes one or more of the possible referents (while the results from the Salience task define a probability distribution over the full set of referents). In the current study, as detailed in the following section, we therefore compare listener behavior across and within context types to predictions from both the RSA model and a baseline literal listener model that combines literal meaning with the prior probability of referring to a referent.

## 3 Current study

The goal of the current study is to investigate whether listeners in one-shot web-based referential communication games make inferences beyond the literal meaning of words by reasoning about rational speakers. In three experiments, which employ the same paradigm as F&G, we investigate the extent to which the pragmatic reasoning assumed by RSA is required to explain human judgments in one-shot web-based communication games. Experiment 1 is a close replication of F&G but systematically examines a broader range of visual context types in order to assess which contexts reliably elicit pragmatic behavior and under what circumstances. Experiment 2 focuses on the role of the prior in pragmatically solvable contexts in order to investigate the effect of the prior on listener judgments and on RSA’s posterior predictions. Experiment 3 increases participant engagement in order to test whether greater engagement elicits more pragmatic responses. In order to test the contribution of RSA’s pragmatic component (the speaker model defined in Eq 3), we evaluate the performance of RSA relative to a baseline literal listener model that is informed by literal word meaning and the prior alone.

### 3.1 Baseline literal listener model

The basic RSA model, as defined in Section 2.2 (Eq 1), is a Bayesian model that calculates the posterior probability that a listener will choose a specific referent by combining a likelihood function that formalizes an informative speaker (the pragmatic component) with a prior probability that reflects empirically measured salience. In order to independently assess the contribution of literal word meaning and speaker informativeness in listener behavior, we contrast the predictions of the basic RSA model with those from a baseline listener model that replaces RSA’s pragmatic component with a truth-condition function that simply determines whether the literal meaning of the given word fits the referent or not. That is, while maintaining RSA’s Bayesian model structure from Eq 1, the likelihood function is replaced with a literal function that simply returns a uniform probability distribution over the words that can be used to refer to the referent:

$$\begin{array}{c} P\left(w|r_{s},C\right)=\frac{⟦w⟧(r_{S})}{\sum_{w^{\prime }\in W}⟦w^{\prime }⟧\left(r_{S}\right)} \end{array}$$ (4)

where ⟦*w*⟧ defines the meaning of word *w* as a function from the set of all possible referents *R* to binary truth values (⟦*w*⟧: *R* → {0, 1}), such that it returns 1 if the meaning of word *w* (e.g., “blue”) fits the referent (e.g., *blue square*), and 0 otherwise (e.g., *green square*), and *W* is defined as the set of words that can be used to refer to referent *r*_*S*_. The baseline literal listener model thus predicts that if multiple objects can be described by a given word, listeners will distribute their choices across those objects, weighted by the prior alone. Although we follow F&G in assuming that words either ‘fit’ an object or not, this may not strictly be the case; see [42] for an approach that relaxes the assumption that semantic truth functions are deterministic.

It should be noted that the formalization of a literal listener model is not new within the context of RSA. In fact, RSA is based on the assumption that the relation between speaker and listener models is recursive: listeners model speakers, who in turn model listeners, etc. The literal listener model (often referred to as *L*_0_) serves as the base case: that is, the informative speaker model from Eq 3 can be formalized as choosing word *w* by maximizing the probability that a naive, literal listener, *L*_0_, would select the correct referent given the literal meaning of *w*. Although some explicit formalizations of *L*_0_ also include a prior, this prior is generally assumed to reflect a uniform distribution in order maintain equivalence to the informative speaker model, as defined by F&G (Eq 3).

As shown in Eq 2, RSA’s formalization of rational speakers includes an *α*-parameter that measures the speaker’s deviation from maximizing utility—in other words, the degree of speaker rationality. In their evaluation of RSA’s performance, F&G set *α* = 1 in order to recover a standard Luce choice rule, but other work has employed parameter-estimation techniques (e.g., [23, 32, 47]), for instance to determine speaker rationality. Indeed, the above defined baseline literal listener model essentially corresponds to the RSA model in which *α* = 0. That is, if *α* < 1, the contribution of the speaker model decreases relative to the contribution of the prior in predicting listener responses. As *α* approximates 0, predictions begin to converge with a literal listener model that is driven by salience. Conversely, for any *α* > 1 the contribution of the speaker model increases, thereby resulting in predictions that diverge more strongly from the baseline model. Hence, the basic RSA model described by F&G (based on Eqs 1 and 3) can be considered a minimal formalization of RSA’s underlying assumption that listeners model informative speakers. Therefore, in order to evaluate the contribution of this pragmatic component of the RSA model in describing listener judgments, we directly compare predictions from the basic RSA model (henceforth, simply ‘RSA’) with predictions from the baseline literal listener model. If we find that the baseline literal listener model outperforms RSA (at *α* = 1), this would indicate that listeners do not reason as pragmatically as previously assumed—since in that case the baseline model will necessarily also outperform RSA at any *α* > 1. If, on the other hand, RSA outperforms the baseline literal listener model, this would indicate that listeners do reason pragmatically about informative speakers.

### 3.2 Broader range of context types

**Fig 3:**
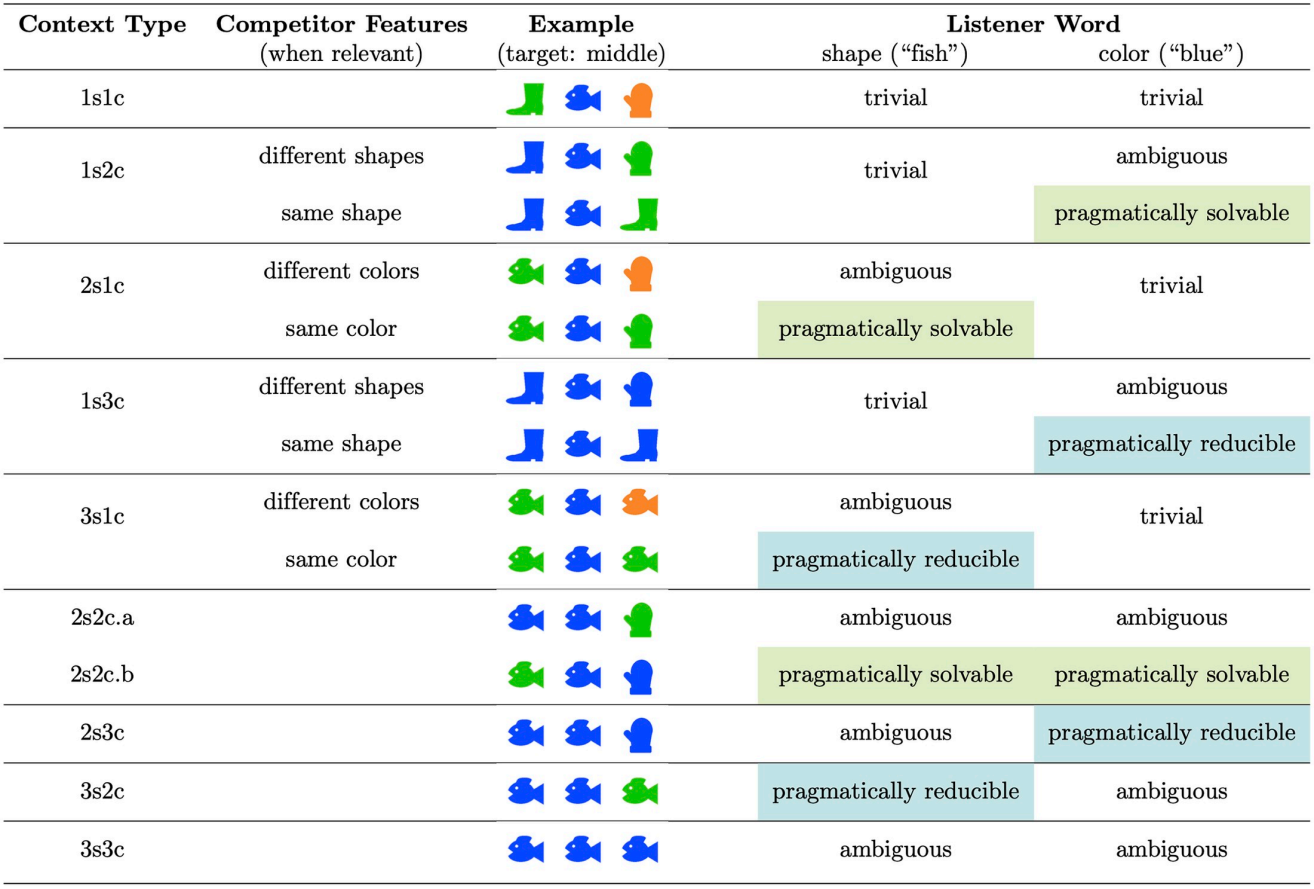
Example items for each of the visual context types used in Experiment 1. The speaker’s target was always in the middle. The first column defines the context type in terms of the number of objects that have target’s shape (s) and color (c). The second column describes the feature overlap between competitors (for those contexts in which it affects RSA’s predictions). The fourth and fifth columns categorize the range of reasoning types in the *Listener* task, given a shape or color word respectively, defining 24 unique conditions (e.g., 1s2c + same shape + color word). Highlighted cells indicate the conditions for which the predictions from RSA and the baseline literal listener model differ: the *pragmatically solvable* conditions (green), and the *pragmatically reducible* conditions (blue). See text for more detail.

The three experiments below systematically explore a larger space of visual contexts than F&G in order to assess which contexts reliably elicit pragmatic behavior and under what circumstances (see Fig 3). We first manipulated the number of competitors that shared color and/or shape with the speaker’s target (i.e., counterbalancing shape and color in the experimental design from F&G; see Fig 2). As mentioned above, F&G observed that RSA makes different predictions in their ‘2.2’ context type depending on how the features are distributed over the objects; therefore we also separated our context type ‘2s2c’ into ‘2s2c.a’ (in which a single competitor shares both color and shape with the target) and ‘2s2c.b’ (in which one competitor shares the target’s shape and the other competitor shares the target’s color), thus resulting in the 10 basic context types shown in the first column of Fig 3.

In addition, however, we found that feature overlap between competitors (Fig 3, second column) also influences the predictions of RSA, depending on the word that the listener observes (i.e., a shape or color word; Fig 3, fourth and fifth columns). Consider, for instance, context type 1s2c (where the speaker’s target has a unique shape and shares its color with one competitor). When given a color word (“blue”), RSA’s predictions will diverge depending on whether the competitors have different shapes or the same shape. When competitor shapes are different, RSA predicts that listeners will distribute their choices equally across the two blue objects (i.e. the intended referent is ambiguous). However, when competitors have the same shape, RSA predicts that a pragmatic reasoner will show a preference for the *blue boot* (i.e. the listener infers that the intended referent is different than the speaker’s actual target). Note that RSA does not make different predictions for this context type when listeners are given a shape word (“fish”) because it uniquely identifies the intended referent. This effect of competitor features on RSA predictions occurs because RSA’s speaker model takes into account the likelihood that a speaker uses a particular word relative to *all* objects in the given context (see Eqs 1 and 3). Therefore, in order to obtain consistent listener predictions for each of our conditions from the RSA model, we manipulated the amount of overlap between the features of the competitors (Fig 3, second column), but only for those combinations of context types and listener words for which this affected the RSA predictions. Combining columns 1 and 2 with a listener word (e.g., 1s2c + same shape + color word) resulted in a total of 24 conditions in which the context types range from three unique objects (Fig 3, first row) to three identical objects (Fig 3, final row).

The highlighted cells in Fig 3 indicate the conditions for which the predictions from RSA and the baseline literal listener model differ. These can be separated into two types of conditions: the *pragmatically solvable* conditions (labeled green in Fig 3; these four conditions are analogous to the critical 2.2b condition in F&G) in which pragmatic reasoning can in principle allow a listener to single out the pragmatic referent, and the *pragmatically reducible* conditions (labeled blue in Fig 3), in which pragmatic reasoning may help in excluding one of the competitor items (see Section 2.3 for a detailed explanation of the pragmatic reasoning involved in these types of conditions). Together, we will refer to these eight conditions as the *pragmatic conditions*. If listeners take into account a rational speaker, then we predict that RSA will provide a better fit to the data than the baseline literal listener model—particularly when only considering the pragmatic conditions. However, if the fit of RSA is no better than the baseline model, this would challenge the conclusion that listeners reason about speakers in these settings.

## 4 Experiment 1: Reevaluating pragmatic reasoning in language games

The goal of Experiment 1 is to systematically examine a broad range of visual context types in order to assess which reliably elicit pragmatic behavior, and under what circumstances. We employ the same general methods as F&G with the following key adjustments: an increased number of trials in the critical pragmatically solvable conditions, and an increased sensitivity to the effects of shape vs color (rather than collapsing across them) while also minimizing the known perceptual salience of color over shape.

### 4.1 Methods

This study was conducted with the approval of the Deutsche Gesellschaft für Sprachwissenshaft (DGfS). All participants gave written consent according to the policies set forth by the Ethics Committee of the DGfS.

#### 4.1.1 Participants

4642 participants were recruited via Amazon’s Mechanical Turk (Mturk; www.mturk.com) crowdsourcing service and were compensated $0.33 for their participation. We restricted our sample to participants with IP addresses located in the United States and who had a minimum 90% approval rating on previous tasks. We used Mturk’s exclusion capabilities (internal tracking of worker ID) to prevent individuals from participating in the experiment more than once. 1268 participants (27:3%) were excluded for the following reasons: 1137 (24:5%) self-identified as non-native or non-fluent English speakers, and a further 118 (2:5%) incorrectly answered one or both of the attention questions. In addition, we excluded 13 participants from the listener task who selected a referent that did not correspond to the literal meaning of the given word. The remaining 3374 participants were randomly assigned to either the Speaker (N = 1143), Listener (N = 1098), or Salience (N = 1133) task. An exit survey revealed the following demographic distributions: *Gender*: *male*(47%), *female*(52%), *other*: (0.004%); *Age*: 18−25(18%), 26−35(39%), 36−45(22%), 46−55(12%), 56−65(7%), *over* 65(2%).

The large proportion of non-native speakers in our sample raises potential concerns about the results from many previous language processing studies using crowdsourcing methods. Many of these studies (including [20, 32, 43]) required participants to have an IP address located in the United States but did not explicitly query participants’ native language. This could be problematic because the U.S. Census reports that more than 20% of people in the United States aged 5 or older speak a language other than English at home, and over 40% of this group speak English less than “very well” [48]. In addition, given the widespread and increasing use of VPNs to mimic IP addresses, it is extremely likely that a significant proportion of workers in such crowdsourcing samples do not actually live in the geographic location reflected by their IP address.

#### 4.1.2 Procedure

The experiment was implemented as a one-shot Mechanical Turk study in which participants were randomly assigned to either the Speaker, Listener, or Salience task. Fig 6 (left; see the S1 File) shows the key screens that participants saw during the experiment. In all tasks, participants were introduced to an interlocutor (“Robert”), who they were told to interact with during the experiment. Then, an attention check was performed to make sure participants were paying attention to the objects displayed. On the critical screen, participants were asked to either pick a word to describe the target object (Speaker task), pick an object based on a word uttered by Robert (Listener task), or pick an object based on Robert saying something incomprehensible (Salience task). Although F&G used a betting paradigm in which participants were asked to distribute bets over a range of options, follow-up studies have shown similar results using forced-choice designs similar to the one used in the current study (e.g., [43]). At the end of the experiment, participants filled out a brief exit survey. For a more detailed description of the experimental procedure, see the S1 File. On average, participants spent 3.7 minutes (*Mdn* = 2.1, *SD* = 6.5 min) on the Human Intelligence Task (HIT), with an average of 16 seconds (*Mdn* = 12, *SD* = 27 sec) on the critical task.

#### 4.1.3 Materials

On the critical screen participants viewed a randomly generated visual context containing three objects. In order to increase the likelihood that participants would consider all of the objects during the task, all speakers were asked to refer to a target object that appeared in the middle position. Each object was assigned a color (blue, green, orange, purple) and a shape (fish, boot, table, mitt). Unique contexts were constructed by systematically manipulating whether one, two, or all three of the objects shared features with the target along one or both of these dimensions, as well as by manipulating the degree of feature overlap between competitor items (see Section 3, Fig 3).

Theoretically, in an ideal set of stimuli for assessing pragmatic reasoning, no single feature would be more salient than another. However, previous work suggests that color may be more perceptually dominant than shape [49, 50], and can therefore lead to pop-out effects. To minimize this potential effect, we strove to reduce the perceptual salience of color by choosing less-saturated chroma from within each principal hue (hexadecimal color codes: blue, 0066FF; green, 00CC00; orange, FF9933; purple, 9933FF). A pilot study tested these chroma on a variety of browser types and with different screen brightness/contrast settings in order to establish that the chroma: (a) were readily identifiable by their given color terms (“blue”, “green”, “orange”,“purple”), (b) were sufficiently distinguishable from each other, and (c) did not differ markedly in terms of salience from each other. The perceptual salience of color over shape was further minimized by choosing iconic shapes that were deemed more interesting than the generic geometric forms (e.g., circles, squares) used in many previous studies.

For each task (Speaker, Listener, Salience), 50 items were randomly generated for each of the 24 conditions from Fig 3, with the constraint that each of the 16 unique shape-color combinations (e.g., blue+ fish) occurred an approximately equal number of times as the speaker’s target. Additionally, for context types in which the objects on the left and right were not identical (e.g., 2s2c.b), items were further counterbalanced such that half had the distribution of features as depicted in their corresponding examples in Fig 3, and half had the mirrored distribution.

#### 4.1.4 Model evaluation / data analysis

In order to obtain model predictions we computed mean behavioral (Speaker, Salience, and Listener) responses separately for each of the 24 conditions described in the previous paragraph. For model evaluations we report the Pearson product-moment correlation (Pearson’s *r*) as well as adjusted *R*^2^ ($R_{adj}^{2}$), *t*, and *p* values. To statistically compare model fits we used the *cocor()* function for comparing overlapping dependent correlations [51] with an alpha of 0.05 in the statistical software package R version 3.6.1 [52] and we report Hittner et al.’s [53] modification of Dunn and Clark’s *z* [54]. Many tests have been proposed for comparing overlapping dependent correlations. For a detailed discussion of these competing tests, see [51] and the references therein. The *cocor()* function computes the results of ten different tests. Although we report Hittner et al.’s [53] *z*, a minimum of eight tests were in agreement for each of the comparisons below.

### 4.2 Results

#### 4.2.1 Speaker task

Participant choices in the Speaker task were highly correlated with RSA’s predictions for informative speakers, formalized in Eq 3 (*r* = 0.93, $R_{adj}^{2}=0.86$, *t* = 11.89, *p* < .0001). This replicates F&G. Indeed, when considering only the context types in which one word (e.g., shape) is more informative than the other (e.g., color), speakers strongly preferred the more informative word regardless of whether it was a shape word (*M* = 0.97, *SD* = 0.02) or a color word (*M* = 0.89, *SD* = 0.05). Overall, however, speakers had a preference for using shape words over color words (shape: 0.59, color: 0.41; exact binomial test: *p* < .0001). This bias was even stronger when considering only those context types (1s1c, 2s2c.a, 2s2c.b and 3s3c) in which shape and color are predicted to be equally likely (shape: 0.70, color: 0.30; *p* < .0001).

Table 2 illustrates these patterns of speaker behavior within the critical pragmatically solvable contexts. When the speaker’s target was the pragmatic referent (e.g., *blue boot*, see condition 2s2c.b), the shape word and the color word are predicted to be equally informative, but participants showed a significant preference for using the shape word over the color word (*p* < .001). However, when the speaker’s target was one of the competitors (e.g., *blue mitt*, see condition 1s2c.ss, or *green boot*, see condition 2s1c.sc), participants clearly preferred to use the more informative word, as predicted by RSA’s informative speaker model, rather than distribute their choices evenly across the two possible literal descriptions (*p*s < .0001).

**Table 2:** Speaker results and an example visual context from the *pragmatically solvable* conditions. Columns represent choice options: color word, shape word. Rows present the distribution of responses when the target was the pragmatic referent (top), color competitor (middle), or shape competitor (bottom).

Target		N	color	shape
pragmatic referent	*blue boot*	91	0.32	0.68
color competitor	*blue mitt*	71	0.06	0.94
shape competitor	*green boot*	60	0.90	0.10

#### 4.2.2 Salience task

Replicating F&G, no correlation between judgments in the Salience and Listener tasks was found (*r* = 0.15, $R_{adj}^{2}=0.01$, *t* = 1.60, *p* = .11). However, one might expect this to be the case due to the large proportion of trials in which the literal meaning of the given word either uniquely identifies the target or excludes one of the competitors. When considering only the pragmatic conditions, the correlation becomes robust (*r* = 0.70, $R_{adj}^{2}=0.48$, *t* = 6.25, *p* < .0001). Further breaking down the pragmatic conditions into pragmatically solvable and pragmatically reducible subtypes reveals that the correlation between Salience and Listener judgments is driven completely by the pragmatically reducible conditions (*r* = 0.95, $R_{adj}^{2}=0.90$, *t* = 12.08, *p* < .0001). No correlation was found for the solvable conditions (*r* = 0.20, $R_{adj}^{2}=0.00$, *t* = 0.98, *p* = .34).

#### 4.2.3 Listener task

Table 3 shows the fit of each model’s predictions to observed listener responses, for different subsets of the data. The models are ranked according to significant fit. The first row shows the overall model fit when all conditions are included in the analyses and predictions of 100% (i.e. when the given word uniquely identifies a referent) and 0% (i.e. when the given word excludes a referent) are included. Although RSA performs well (*r* = 0.87), replicating F&G, it does not provide as good a fit as the baseline literal listener model. A comparison of overlapping dependent correlations reveals that the baseline performs significantly better than RSA (*z* = 3.17, *p* < .01). The second row in Table 3 shows the model fits after removing predictions of 100% and 0%, which provide a more conservative assessment of model fit (therefore all subsequent correlation results will exclude such predictions). Although the fit of both models decreases, the rankings do not change because the models agree on predictions for 100 and 0. Thus, the baseline still performs significantly better than RSA (*z* = 3.24, *p* < .01).

**Table 3:** Experiment 1. Model evaluation results from the *Listener* task comparing RSA to the baseline literal listener model (LL). Models are ranked according to significant fit. cocor- *p*: *p*-value for comparison of overlapping dependent correlations.

Dataset	Rank	Model	*r*	$R_{adj}^{2}$	*t*	*p*	cocor-*p*
Overall (including estimates of 0 & 100)	1	LL	0.89	0.79	20.80	< .0001	} < .01
	2	RSA	0.87	0.76	18.82	< .0001	
Overall	1	LL	0.61	0.36	6.12	< .0001	} < .01
	2	RSA	0.52	0.25	4.81	< .0001	
Pragmatic conditions	1	LL	0.84	0.69	8.41	< .0001	} < .001
	2	RSA	0.69	0.46	5.27	< .0001	
Pragmatically reducible conditions	1	LL	0.96	0.91	12.46	< .0001	} < .01
	2	RSA	0.92	0.83	8.46	< .0001	
Pragmatically solvable conditions	–	LL	−0.12	−0.06	−0.47	.65	} .36
	–	RSA	−0.27	0.00	−1.03	.32	

As a stronger test of model fit we then conducted model evaluations on the pragmatic conditions. Recall that these are the conditions which are in principle amenable to pragmatic reasoning and for which the models make different predictions (see Section 3). The results for the pragmatic conditions in Table 3 indicate that the baseline literal listener model performs significantly better than RSA on these conditions (*z* = 3.39, *p* < .001). Further subdividing the pragmatic conditions into the pragmatically reducible and pragmatically solvable conditions reveals important differences between these two condition types. Results for pragmatically reducible conditions in Table 3 show that both RSA and the baseline literal listener model provide excellent fits in these conditions. Note, however, that this excellent fit is precisely because both models choose the (non-pragmatic) competitor over the pragmatic referent. Indeed, this preference appears to be driven by the overwhelming salience of the competitor, which listeners strongly prefer regardless of whether the given word was a color or shape word (Table 4). Further evidence in support of this conclusion comes from comparing the fit of RSA to a version of RSA that assumes a uniform prior, which allows us to quantify the contribution of the prior to RSA’s posterior probability. When considering only the pragmatic conditions, results indicate that the fit of the uniform prior version of RSA not only decreased markedly relative to RSA, but also reversed direction (*r* = −0.21, $R_{adj}^{2}=0.01$, *t* = −1.15, *p* = 0.26). Moreover, when the same analysis is conducted on the subset of pragmatically reducible conditions, the fit of the uniform prior version of RSA completely reverses direction (*r* = −0.99, $R_{adj}^{2}=0.97$, *t* = −21.03, *p* < .0001). This pattern of results suggests that the prior largely overrides incorrect predictions generated by the pragmatic component of the RSA model. Although these findings show that RSA is flexible enough to allow the prior to dominate over the speaker model (the pragmatic component) in determining the posterior—when the speaker model’s preferences are weak and one object is deemed to be particularly salient—we nevertheless find that the baseline literal listener model still performs better (*z* = 2.93, *p* < .01).

**Table 4:** Experiment 1. Behavioral results and two example visual contexts from the *pragmatically reducible* conditions. Examples differ depending on whether the given Listener word was a *shape* word (upper panel) or *color* word (lower panel). Columns represent choice options: pragmatic referent 1 (left-most pragmatic referent), pragmatic referent 2, and competitor. Rows in each panel present the distribution of responses in the *Salience* task (top row) and *Listener* task (bottom row). Salience responses are calculated separately over the contexts in which the competitor has a unique shape vs color.

Task	N	pragmatic ref.1	pragmatic ref.2	competitor
		*blue boot*	*blue boot*	*blue mitt*
Salience	90	0.12	0.19	0.69
Listener—“blue”	90	0.12	0.09	0.79
		*blue boot*	*blue boot*	*green boot*
Salience	99	0.10	0.13	0.77
Listener—“boot”	90	0.13	0.10	0.77

Finally, the solvable contexts are perhaps the most critical subset of conditions, since they serve as the classic example of pragmatic reasoning in reference games. The lower panel of Table 3 indicates that neither RSA nor the baseline model provide a good fit to listener responses in these conditions. Behavioral results for the solvable conditions (Table 5) reveal that in contrast to both F&G and [32], the distribution of Salience responses in the current experiment is largely uniform across all three objects (exact multinomial test: all *p*s >0.23). In addition, Listener responses are split evenly over the two objects that can be described by the given word, regardless of whether it was a color word or shape word (exact binomial tests: color: *p* = 1; shape: *p* = .46). This pattern of results suggests that neither pragmatic considerations nor the prior drove listeners’ choices. In other words, participants simply appear to be at chance in guessing at the speaker’s intended referent.

**Table 5:** Experiment 1. Behavioral results and an example visual context from the *pragmatically solvable* conditions. Columns represent choice options: pragmatic referent, color competitor, and shape competitor. Rows present the distribution of responses in the *Salience* task (top row) and *Listener* task when given a color or shape word (bottom rows).

Task	N	pragmatic ref.	color competitor	shape competitor
		*blue boot*	*blue mitt*	*green boot*
Salience	188	0.37	0.36	0.27
Listener—“blue”	97	0.49	0.51	0.00
Listener—“boot”	90	0.46	0.00	0.54

### 4.3 Discussion

We set out to systematically examine a broad range of visual context types with two goals in mind: (1) to assess which contexts reliably elicit pragmatic behavior in this task, and under what circumstances, and (2) to evaluate the performance of RSA by contrasting its predictions with that of a plausible baseline literal listener model that does not assume pragmatic reasoning. To do so, we used the same general methods as F&G but increased the number of trials in the critical pragmatically solvable conditions and increased our sensitivity to the effects of shape versus color.

For the Speaker task, we replicated the result from F&G showing that speaker choices were highly correlated with RSA’s predictions for informative speakers. This finding demonstrates that participants are able to determine the most informative word for a given referent in these settings. This thus offers empirical support for RSA’s assumption to incorporate an informative speaker model as a likelihood estimate. In addition, we found that speakers had a preference for using nouns (shape words) over adjectives (color words) when we considered only those contexts where nouns and adjectives were equally informative. These findings replicate the behavioral results from [32], who argue that this effect arises because it is more natural to use nouns than adjectives when referring to concrete objects (see also the discussion in Section 7.1).

In the Listener task, we replicated F&G by showing that RSA provided a good fit to the overall data set, as well as to the subset of pragmatic conditions. However, when examining the two terms that are integrated to compute RSA’s posterior predictions—the pragmatic component (i.e. the model of an informative speaker) and the prior (as measured by the Salience task)—we found that the prior largely overrode the predictions of RSA’s pragmatic component. In fact, the baseline literal listener model performed significantly better than RSA in both of these analyses. Interestingly, in the critical pragmatically solvable conditions (which are analogous to the 2.2b condition in F&G), no significant differences were found between listener responses for the pragmatic referent and its competitor, regardless of whether listeners observed a color or shape word. Put differently, listeners appeared to be at chance in choosing between the two objects that matched the literal meaning of the given word. As a result, neither RSA nor the baseline listener model provided a good fit to this subset of the data. Thus, we find no evidence that listeners used pragmatic reasoning to resolve the speaker’s intended referent in the current task—not even when considering only the conditions for which pragmatic reasoning could in principle make a difference.

The pattern of behavioral results found here differs from [32], who specifically investigated pragmatically solvable contexts. In contrast to our results, they found evidence of pragmatic behavior when listeners were given a color word, but not when they were given a shape word (Table 1). One possible explanation for these differences in listener results across the two studies is the role of the prior in listener responses. In the current experiment, Salience judgments were more uniformly distributed across all three objects, while in [32] the shape competitor was judged to be almost five times more salient than the pragmatic referent. This disparity may explain why listeners in [32] appeared to abandon pragmatic reasoning in the shape word condition. Experiment 2 will directly investigate this potentially critical role of the prior by focusing on pragmatically solvable contexts.

In summary, the behavioral results suggest that while speakers are informative in the current paradigm, listeners do not seem to reason pragmatically about this aspect of speaker behavior. Moreover, despite the high correlation of the RSA model’s posterior predictions to listener responses, RSA did not perform better than a baseline literal listener model. This indicates that the close fit of RSA to the overall data set was driven by a combination of two factors: (1) the simpler conditions, in which no pragmatic reasoning is required, and (2) the prior, which overrode the predictions of RSA’s pragmatic component in the pragmatically reducible contexts. In order to better understand these results, Experiment 2 focuses on the pragmatically solvable conditions and investigates the extent to which listener behavior can be explained in terms of RSA’s two components: the informative speaker model and the prior probability that an object will be referred to.

## 5 Experiment 2: The role of the prior in *pragmatically solvable* contexts

The finding from Experiment 1 that listeners were at chance in the pragmatically solvable conditions is surprising given that solvable contexts serve as the canonical example of pragmatic inference in referential communication games. Moreover, this finding is at odds with previous work, which has shown that listeners can—at least under certain circumstances—respond pragmatically in such contexts [32]. One explanation for these mixed results may have to do with the role of the prior, as measured empirically by the Salience task: in Experiment 1, Salience judgments were uniformly distributed across all three objects, while participants in [32] (which used geometric stimuli similar to those used by F&G) deemed the shape competitor to be most salient and the pragmatic referent to be the least salient. To better understand the role of the prior in predicting Listener responses, we conducted a second experiment that focuses on the pragmatically solvable conditions and investigates the factors that underlie Salience judgments in more detail. More specifically, we investigate the extent to which the following features may contribute to Listener and/or Salience responses: stimulus type (iconic, geometric), an object’s position (left, middle, right), an object’s shape, an object’s color, whether the object has a unique shape, and whether the object has a unique color. As in Experiment 1, we evaluate the performance of RSA against the baseline literal listener model.

### 5.1 Methods

This study was conducted with the approval of the Deutsche Gesellschaft für Sprachwissenshaft (DGfS). All participants gave written consent according to the policies set forth by the Ethics Committee of the DGfS.

#### 5.1.1 Participants

1671 participants were recruited via Amazon’s Mechanical Turk using the same criteria as in Experiment 1. 231 participants (13.8%) were excluded for the following reasons: 142 (8.5%) self-identified as non-native or non-fluent English speakers, 77 (4.6%) incorrectly answered one or both of the attention questions, and 12 (0.7%) participants in the listener task selected a referent that did not correspond to the literal meaning of the given word. The remaining 1440 participants were randomly assigned to either the Listener (N = 960) or Salience (N = 480) task. Experiment 1 already showed that speakers are informative in this paradigm, thus no Speaker task was run in Experiment 2. An exit survey revealed the following demographic distributions: Gender: male (58%), female (42%), other: (0%); Age: 18-25 (14%), 26-35 (39%), 36-45 (23%), 46-55 (14%), 56-65 (8%), over 65 (0%).

#### 5.1.2 Procedure

The procedure for Experiment 2 was identical to Experiment 1, with one exception. In order to establish a baseline for how interesting and engaging participants find the one-shot web-based paradigm, the exit survey asked an additional question: “How engaging did you find this HIT?” (see the S1 File). On average, participants spent 3.9 minutes (*Mdn* = 1.9, *SD* = 5.0 min) on the HIT, with an average of 16 seconds (*Mdn* = 12, *SD* = 30 sec) spent on the critical task.

#### 5.1.3 Materials

**Fig 4:**
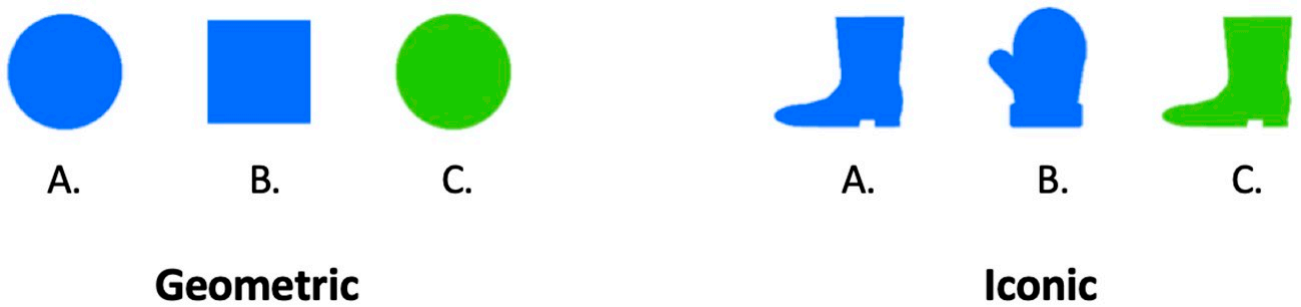
Experiment 2. Example visual contexts for *geometric* and *iconic* stimulus types.

A 2 (stimulus type) x 2 (shape) x 2 (color) design was used to construct parallel sets of pragmatically solvable visual contexts (Fig 4). Stimulus type was either geometric or iconic. Two shapes were chosen for each stimulus type: geometric (circle, square), iconic (boot, mitt). To control for the effect of color, two colors (blue, green) were selected from Experiment 1 and used for both stimulus types. This resulted in four shape-color combinations for each stimulus type, which were yoked such that wherever one appeared in the geometric stimuli (e.g., blue+ circle), its counterpart appeared in the iconic stimuli (e.g., blue+ boot). Twenty-four fully counterbalanced visual contexts were created for each stimulus type such that each of the four shape-color combinations occurred an equal number of times as the pragmatic referent and an equal number of times in each position (left, middle, right). As this was a one-shot experiment, we increased power for detecting the factors underlying behavioral judgments by randomly assigning ten participants to the Salience task for each of the 48 visual contexts described above. Similarly, in the Listener task each of the 48 visual contexts was presented with either a color or shape word that could be used to refer to the pragmatic referent and ten participants were randomly assigned to each of these 96 context+word conditions.

### 5.2 Results

#### 5.2.1 Behavioral results

*5.2.1.1 Salience task*. To investigate the factors that drive Salience responses, we conducted linear regression analyses to assess whether particular features of the stimuli were significant predictors of an object’s salience. We first investigated whether results differed across stimulus type, comparing the *geometric* stimuli used by F&G to the *iconic* stimuli used in Experiment 1. Results showed that stimulus type did not predict Salience responses (*p* = 1). However, a closer look at the Salience responses per stimulus type (Table 6) seems to suggest that the geometric stimuli actually result in a stronger bias towards the shape competitor (i.e., the object with a unique color) than the iconic stimuli used in Experiment 1. This bias was confirmed by exact multinomial tests, which revealed that for the geometric stimuli, the shape competitor (e.g., *green circle*) was deemed more salient than both the pragmatic referent (e.g., *blue circle*) (*p* < .05) and the color competitor (e.g., *blue square*) (*p* < .01). Salience responses for the pragmatic referent and the color competitor did not differ (*p* = .34). In contrast, Salience responses for the iconic stimuli were uniformly distributed across the three objects (all *p*s > .3), replicating the Salience results for the pragmatically solvable conditions in Experiment 1. Thus, stimulus type did not predict Salience responses, however these findings suggest that the iconic stimuli provide a better test of pragmatic reasoning in reference games than the geometric stimuli because the former do not exhibit a color pop-out effect.

**Table 6:** Experiment 2. Behavioral results and example visual contexts for the geometric stimuli (top panel) and the iconic stimuli (bottom panel). The third column identifies the total number of responses in a given task (Salience/Listener–color word/Listener–shape word). The final three columns show the percentage of responses over the object types (pragmatic referent/color competitor/shape competitor).

Stimuli	Task	N	pragmatic ref.	color comp.	shape comp.
Geometric			*blue circle*	*blue square*	*green circle*
	Salience	240	0.30	0.25	0.44
	Listener—“blue”	240	0.56	0.44	0.00
	Listener—“circle”	240	0.45	0.00	0.55
Iconic			*blue boot*	*blue mitt*	*green boot*
	Salience	240	0.35	0.30	0.35
	Listener—“blue”	240	0.64	0.36	0.00
	Listener—“boot”	240	0.45	0.00	0.55

To investigate whether multiple factors interacted to influence Salience judgments, a regression-tree analysis was conducted based on six object features: stimulus type (geometric, iconic), shape (circle, square, boot, mitt), color (blue, green), position (left, middle, right), shape uniqueness (T, F), and color uniqueness (T, F). Results showed that the position of the object was the only factor that survived 10-fold cross-validation: objects in the middle were deemed to be approximately 16% more salient than objects on the left or right. A linear regression analysis with position as its only feature confirmed the significance of position as a predictor for salience (*R*^2^ = 0.17, *F*(2, 141) = 15.13, *p* < .001).

*5.2.1.2 Listener task*. As in the Salience task, linear regression analyses revealed that stimulus type was not a reliable predictor of listener behavior in either the color word (*p* = 1) or shape word (*p* = 1) subsets. These results were confirmed by decision tree analyses. Thus, for the sake of brevity and in order to maintain comparability of the current results to Experiment 1, the remainder of this section focuses on the iconic stimuli only.

Table 6 shows that upon hearing a color word (e.g., “blue”), listeners had a preference for the pragmatic referent when viewing iconic stimuli (*p* < .0001). This preference is consistent with the behavior of an ideal pragmatic listener (see Section 2.3) and replicates the listener results for color words found by [32] (Table 1). Thus, this finding does not replicate the approximately 50/50 distribution of choices between the pragmatic referent and the color competitor found in Experiment 1. In contrast, when given a shape word, listener choices showed a slight numerical preference for the shape competitor, but this did not reach significance (*p* = .18).

**Fig 5:**
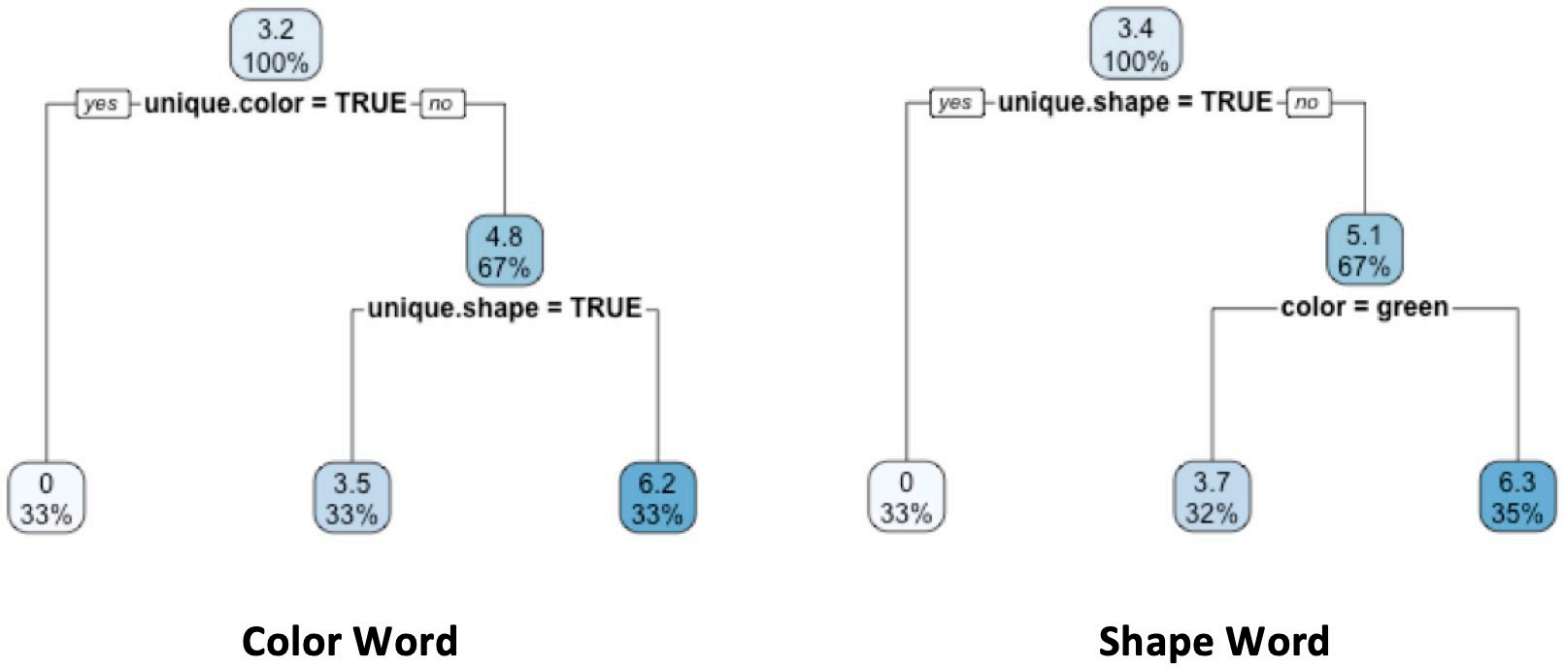
Experiment 2. Regression tree results for the *Listener* task for the iconic stimuli only. *Left*: Color word condition. *Right*: Shape word condition. Each node shows the predicted number of responses (out of 10) and the percentage of observations in the node.

To identify how listener behavior differed across the color word and shape word conditions, a regression tree analysis was conducted on the color word and shape word subsets of the listener data separately. The analysis included the above described predictors (stimulus type, shape, color, position, shape uniqueness, color uniqueness), as well as the participants’ Salience ratings for the object as part of the given visual scene. Fig 5 presents the final decision trees after 10-fold cross-validation. Note that the top branching node in both decision trees (resulting in 0 predicted responses in the left-most terminal node) simply captures the fact that the literal meaning of the given word always excludes one object: in the pragmatically solvable conditions, words presented to the listener always identify overlapping properties, which means that a color word (“blue”) always excludes the object with a unique color (i.e., the shape competitor in Table 6). Similarly, a shape word (“boot”) always excludes the object with a unique shape (i.e., the color competitor in Table 6). For the color word subset (left tree in Fig 5), the second branching node determines whether an object has a unique shape, thus distinguishing between the color competitor (unique shape) and the pragmatic referent (no unique shape): the regression-tree model predicts that the color competitor will only be chosen 3.5/10 times, whereas the pragmatic referent will be chosen 6.2/10 times. This decision is consistent with the predictions of the pragmatic component of the RSA model. For the shape word subset (right tree in Fig 5), the second node determines the object’s color: blue objects are predicted to be selected more often (6.3/10 times) than green objects (3.7/10 times). This is a non-pragmatic decision and thus provides no evidence that listeners make inferences beyond the literal meaning of shape words.

To investigate whether the results of the Salience task can help to clarify the above findings, we tested whether Salience responses were correlated with Listener responses. We observed a weak and marginally significant correlation (*r* = 0.16, $R_{adj}^{2}=0.02$, *t* = 1.97, *p* = .05), which was driven by the shape word condition (*r* = 0.24, $R_{adj}^{2}=0.05$, *t* = 2.11, *p* < .05). This likely reflects the effect of the *color* decision node in the regression-tree analyses above. Furthermore, linear regression analyses also showed that both the object’s color (*R*^2^ = 0.06, *F*(1, 70) = 5.24, *p* < .05) and its salience (*R*^2^ = 0.05, *F*(1, 70) = 4.44, *p* < .05) were weak but reliable predictors of Listener responses in the shape word condition. Salience and Listener responses were not correlated in the color word condition (*p* = .47).

#### 5.2.2 Model evaluation

In order to obtain model predictions we computed mean behavioral (Salience and Listener) responses separately for each of the 48 visual contexts described in Section 5.1. Table 7 shows the fit of RSA and the baseline literal listener model to listener responses (after removing predictions for 0 and 100). When evaluating the entire data set (i.e., when both iconic and geometric stimulus types are included in the analysis; upper rows), RSA performs numerically better than the baseline model, but this difference is not significant (*z* = −0.90, *p* = .37). When analyzing the color and shape word conditions separately, we can see that RSA performs significantly better than the baseline for the color word condition (*z* = −2.89, *p* < .01), but the baseline model performs significantly better than RSA for the shape word condition (*z* = 2.17, *p* < .05). Because stimulus type was not a reliable predictor of listener responses, we again focus on the iconic stimuli (highlighted rows). Here the same pattern holds as above, but only the color word condition reaches significance (color word condition: *z* = −2.65, *p* < .01; shape word condition: *z* = 1.45, *p* = .15). For completeness, we also include the results for the geometric stimuli (bottom rows). Again, the same pattern holds but the differences between models do not reach significance (color word condition: *z* = −1.36, *p* = .17; shape word condition: *z* = 1.34, *p* < .18).

**Table 7:** Experiment 2. Model evaluation results from the *Listener* task comparing RSA to the baseline literal listener model (LL). All correlations tests exclude predictions for 0 and 100. Models are ranked according to significant fit. cocor- *p*: *p*-value for comparison of overlapping dependent correlations. The text focuses on the iconic stimuli (highlighted rows) because stimulus type was not a reliable predictor of listener responses.

Dataset	Listener word	Rank	Model	*r*	$R_{adj}^{2}$	*t*	*p*	cocor-*p*
Overall	color / shape word	–	RSA	0.30	0.09	4.13	< .0001	}.37
		–	LL	0.28	0.07	3.75	< .001	
	color word	1	RSA	0.40	0.15	3.89	< .001	} < .01
		2	LL	0.26	0.06	2.43	< .05	
	shape word	1	LL	0.30	0.08	2.92	< .01	} < .05
		2	RSA	0.22	0.04	2.14	< .05	
Iconic stimuli	color word	1	RSA	0.45	0.18	3.13	< .01	} < .01
		2	LL	0.28	0.05	1.78	.08	
	shape word	–	LL	0.15	0.00	0.99	.33	} .15
		–	RSA	0.08	−0.02	0.48	.64	
Geometric stimuli	color word	–	RSA	0.34	0.09	2.31	< .05	}.17
		–	LL	0.24	0.04	1.62	.11	
	shape word	–	LL	0.43	0.16	3.18	< .01	}.18
		–	RSA	0.37	0.12	2.66	< .05	

As in Experiment 1, in order to quantify the contribution of the prior to RSA’s posterior probability, we compared the fit of RSA to a uniform prior version of RSA. Results revealed that for the color word conditions RSA did not perform as well as the uniform prior version of RSA (overall: *r* = 0.44, $R_{adj}^{2}=0.18$, *t* = 4.44, *p* < .0001; iconic: *r* = 0.56, $R_{adj}^{2}=0.30$, *t* = 4.19, *p* < .001). This finding suggests that incorporating the prior into the model hinders RSA’s performance when given a color word. In contrast, for the shape word conditions, relative to RSA the fit of the uniform prior version of RSA decreased and reversed direction (overall: *r* = −0.24, $R_{adj}^{2}=0.05$, *t* = −2.31, *p* < .05; iconic: *r* = −0.20, $R_{adj}^{2}=0.01$, *t* = −1.26, *p* = .22), replicating Experiment 1. This finding suggests, as observed in Experiment 1, that the prior largely overrides incorrect predictions generated by the pragmatic component of the RSA model.

#### 5.2.3 Engagement

A possible criticism of the one-shot web-based method for testing pragmatic reasoning is that it is not very interactive and therefore may not allow for pragmatic enrichments to happen in full. In order to asses how engaging the current paradigm actually is, we directly asked participants in the exit survey to rate how engaging they found the HIT on a scale from 0 (*not at all*) to 100 (*very much*). Results showed that participants found the task to be quite engaging (*M* = 81.4, *SD* = 21.1). This will serve as our baseline for Experiment 3, where we test whether increasing participant engagement leads to more pragmatic behavior.

### 5.3 Discussion

In order to determine whether the conflicting results between Experiment 1 and previous findings [20, 32] may have been due to differences in stimulus type, Experiment 2 included both iconic and geometric stimuli. Behavioral results from both the Salience and Listener tasks revealed that stimulus type did not affect responses, showing that the behavioral results in Experiment 1 were not driven by some specific feature of the iconic stimuli. Instead, behavioral results in the Salience task revealed that object position was the unique factor influencing Salience judgments, with the middle position being most salient.

In contrast to the results from Experiment 1, behavioral responses in the Listener task showed evidence of pragmatic reasoning when given a color word; i.e. listeners preferred the pragmatic referent over the color competitor. However, we found no evidence of pragmatic reasoning when listeners were given a shape word; there was a non-significant numerical preference for the shape competitor over the pragmatic referent (in line with Experiment 1). This pattern of effects is similar to [32], who also found pragmatic behavior in the color word condition and non-pragmatic behavior in the shape word condition. Note that the prior is unable to explain this asymmetry across color and shape conditions, as Salience judgments do not differ across these conditions. Interestingly, a closer look at the Listener responses showed that in the shape word condition, color was a significant predictor—suggesting that in at least some cases, choices for the pragmatic referent in the shape word condition may have been driven by the relative salience of the pragmatic referent rather than by pragmatic reasoning about an informative speaker.

Model evaluation results confirm that RSA performed better than the baseline literal listener model in the color word condition, indicating that listeners can reason pragmatically in this paradigm. In the shape word condition, however, the baseline model outperformed RSA, indicating that listeners do not necessarily default to pragmatic reasoning when given the opportunity. Note, however, that while these fits are an improvement over the performance of both models for the pragmatically solvable conditions in Experiment 1 (where neither model’s predictions correlated with Listener judgments; this was also true when considering the color word and shape word conditions separately), the correlations are still quite modest when compared to the correlations reported in F&G for their overall data set.

In sum, Experiment 2 finds only modest evidence of pragmatic reasoning, and only in the color word condition. One possible explanation is that the one-shot web-based paradigm may not be sufficiently engaging to allow for pragmatic enrichments to happen in full. This hypothesis is tested in Experiment 3.

## 6 Experiment 3: Increasing listener engagement

In the previous two experiments, we found modest evidence for pragmatic reasoning in language games, which seem limited to conditions in which listeners observed a color word. We hypothesize that this finding may be due to the artificial nature of the one-shot web-based paradigm. Experiment 3 tests this hypothesis by creating a situation in which listeners should be highly motivated to correctly infer the speaker’s intended referent. To this end, we designed a version of the study that attempted to increase participant interactivity as much as possible while maintaining all other aspects of the previous design. Participants were told, “The city you live in is in great danger! The super villain Dr. Sinisterioso has planted a bomb and it is about to go off!” Participants were then instructed that their task was to communicate with a remote partner in order to disarm the bomb (described in more detail below). In addition to this engagement manipulation, the visual contexts were expanded to include all conditions where RSA and the baseline literal listener model make different predictions. This includes both the pragmatically solvable conditions from Experiment 2, as well as the pragmatically reducible conditions from Experiment 1.

### 6.1 Methods

This study was conducted with the approval of the Deutsche Gesellschaft für Sprachwissenshaft (DGfS). All participants gave written consent according to the policies set forth by the Ethics Committee of the DGfS.

#### 6.1.1 Participants

1175 participants were recruited via Amazon’s Mechanical Turk using the same criteria as in Experiment 1. 364 participants (31.0%) were excluded for the following reasons: 265 (22.6%) self-identified as non-native or non-fluent English speakers, 96 (8.2%) incorrectly answered one or both of the attention questions, and three (0.3%) participants in the Listener task selected a referent that did not correspond to the literal meaning of the given word. Each of the remaining 811 participants was randomly assigned to either the Listener (N = 405) or Salience (N = 406) task. As in Experiment 2, no Speaker task was run in this experiment. An exit survey revealed the following demographic distributions: Gender: male (56%), female (44%), other: (0%); Age: 18-25 (16%), 26-35 (48%), 36-45 (21%), 46-55 (10%), 56-65 (4%), over 65 (0%).

#### 6.1.2 Procedure

**Fig 6:**
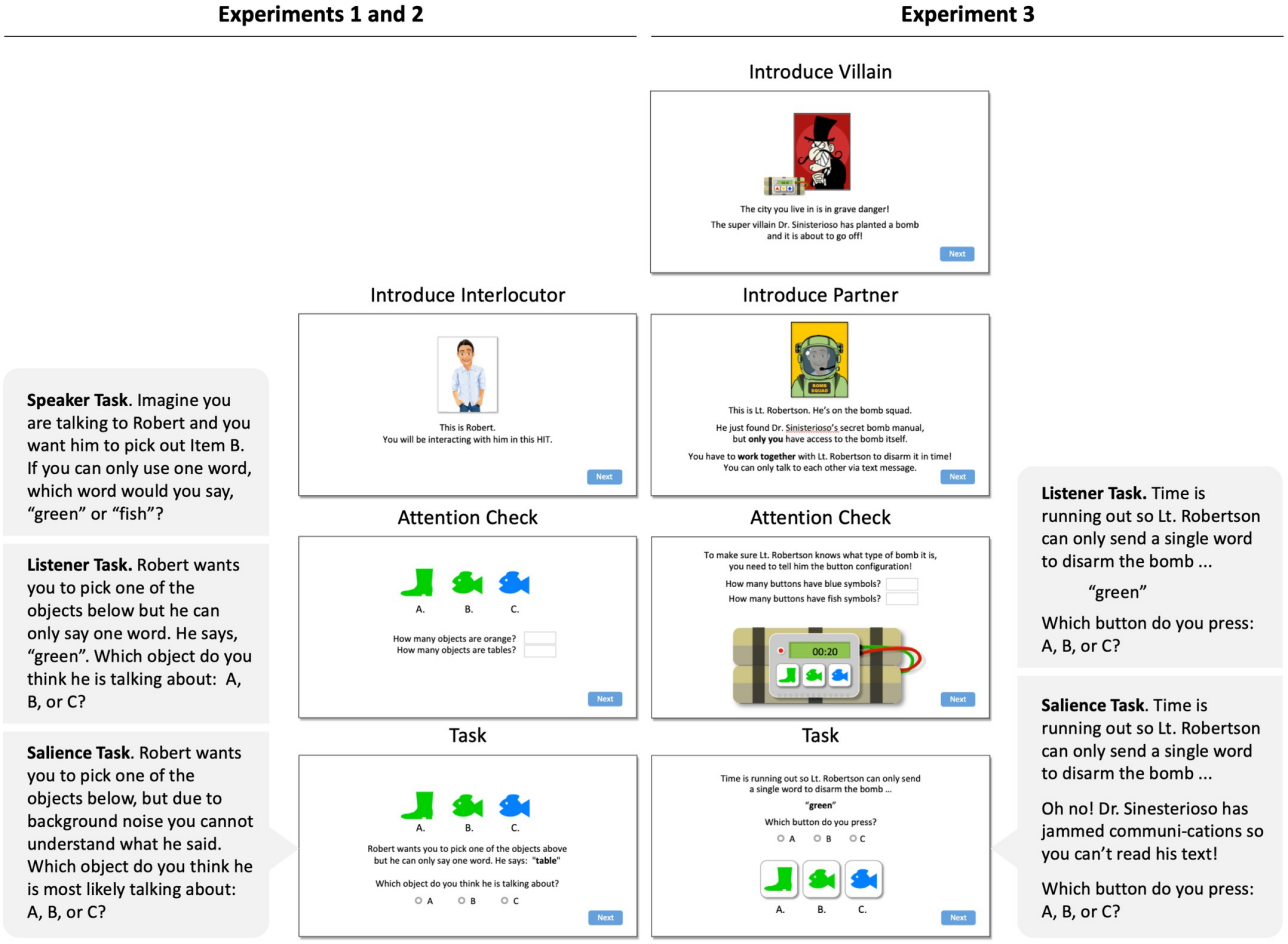
Key screens in Experiments 1 and 2 (left) and Experiment 3 (right).

The procedure for Experiment 3 was similar to Experiments 1 and 2, except for the introductory text and formulation of the task, which was designed to be more engaging than the previous studies. The key screens for Experiment 3 are shown in Fig 6 (right). Instead of simply introducing an interlocutor (as in the previous studies), participants were presented with a short background story about a super villain who had planted a bomb in the city. The interlocutor is now a member of the bomb squad (Lt. Robertson) who will help the participant to disarm the bomb. There was no explicit time pressure during the task, but the background story was presented in such a way that the participant’s response seemed to be a matter of urgency. In addition, the exit survey included the same engagement question as in Experiment 2. The full experimental procedure is described in the S1 File. On average, participants spent 5.5 minutes (*Mdn* = 2.5, *SD* = 8.7 min) on the HIT, with an average of 13 seconds (*Mdn* = 11, *SD* = 8 sec) spent on the critical task.

#### 6.1.3 Materials

The results of Experiment 2 suggested that the iconic stimuli from Experiment 1 provide a better test of pragmatic reasoning in reference games than the generic geometric stimuli used in previous studies, because the former do not exhibit a color pop-out effect. Therefore, we used the same materials as in Experiment 1 (see Fig 3). However, we only tested a subset of the conditions, namely those in which RSA and the baseline literal listener model make different predictions (the conditions highlighted in green and blue in Fig 3). As in Experiment 1, 50 items were randomly generated for each of the eight conditions that were presented, again with the constraint that each of the 16 unique shape-color combinations occurred an approximately equal number of times as the speaker’s target. These items were tested in the both the Listener and Salience tasks.

### 6.2 Results

#### 6.2.1 Engagement

Exit survey results showed that participants found the current version of the HIT to be more engaging (*M* = 87.6, *SD* = 17.6) than the more generic version used in Experiment 2 (*M* = 81.4, *SD* = 21.1; *t*(1932) = −7.51, *p* < .0001). This finding indicates that our attempt to increase participant engagement was successful.

#### 6.2.2 Behavioral results

*6.2.2.1 Pragmatically reducible conditions*. Table 8 presents the behavioral results for the pragmatically reducible conditions. As in Experiment 1, participants in the Salience task had a clear preference for the competitor, which was the only object with a unique feature. Results showed that the competitor was significantly more salient than both the left-most pragmatic referent (exact multinomial test: *p* < .0001) and the second pragmatic referent (*p* < .0001). Salience ratings for the two pragmatic referents did not differ (*p* = .87). In the Listener task, RSA predicts that listeners will infer that the speaker intended to refer to one of the two identical pragmatic referents. However, in both the color word and shape word conditions, listeners overwhelmingly preferred the highly salient competitor object (all *p*s < .0001). These findings replicate the non-pragmatic behavior of listeners for the pragmatically reducible conditions in Experiment 1.

**Table 8:** Experiment 3. Behavioral results and two example visual contexts from the *pragmatically reducible* conditions. Examples differ depending on whether the given Listener word was a *shape* word (upper panel) or *color* word (lower panel). Columns represent choice options: pragmatic referent 1 (left-most pragmatic referent), pragmatic referent 2, and competitor. Rows in each panel present the distribution of responses in the *Salience* task (top row) and *Listener* task (bottom row). Salience responses are calculated separately over the contexts in which the competitor has a unique shape vs color.

Task	N	pragmatic ref.1	pragmatic ref.2	competitor
		*blue boot*	*blue boot*	*blue mitt*
Salience	105	0.07	0.13	0.80
Listener—“blue”	103	0.17	0.10	0.73
		*blue boot*	*blue boot*	*green boot*
Salience	99	0.11	0.12	0.77
Listener—“boot”	102	0.04	0.08	0.88

*6.2.2.2 Pragmatically solvable conditions*. Table 9 presents the behavioral results for the pragmatically solvable conditions. Salience responses were uniformly distributed across the three objects (all *p*s > .16), replicating the Salience results for pragmatically solvable conditions in both Experiments 1 and 2. Listener results show that participants in the color word conditions had a clear preference for the pragmatic referent (*p* < .05). This finding replicates the pragmatic behavior found for color words in Experiment 2 and [32], and suggests that the 50/50 distribution of choices between the pragmatic referent and the color competitor found in Experiment 1 was spurious. In contrast, despite the increased motivation listeners were given in the current study to correctly interpret the speaker’s intended referent, listeners showed a significant preference for the shape competitor when given a shape word (*p* < .01). This finding is consistent with [32], who also found a clear preference for the shape competitor, and indicates that participants did not go beyond the literal meaning of the given shape word. Note once again that the prior is unable to explain this asymmetry between color word and shape word conditions, as Salience responses are the same in both cases.

**Table 9:** Experiment 3. Behavioral results and an example visual context from the *pragmatically solvable* conditions. Columns represent choice options: pragmatic referent, color competitor, and shape competitor. Rows present the distribution of responses in the *Salience* task (first row) and *Listener* task when given a color or shape word (bottom rows).

Task	N	pragmatic ref.	color competitor	shape competitor
		*blue boot*	*blue mitt*	*green boot*
Salience	202	0.27	0.38	0.35
Listener—“blue”	99	0.61	0.39	0.00
Listener—“boot”	101	0.37	0.00	0.64

#### 6.2.3 Model evaluation

Table 10 shows the fit of each model’s predictions to observed listener responses after removing predictions for 0 and 100. On the overall data set, both models perform very well (*r*s >0.79). Although the baseline literal listener model has a numerically better fit than RSA, this difference did not reach significance (*z* = 0.34, *p* = .73). However, RSA performs numerically better for the color word condition (*z* = −1.42, *p* = .15), while the baseline model performs significantly better for the shape word condition (*z* = 2.03, *p* < .05). When we consider the pragmatically solvable and reducible conditions separately, we can see that the good fits in the overall data set are driven by the pragmatically reducible conditions, where the baseline model performs marginally better than RSA for both the color word (*z* = 1.83, *p* = .07) and shape word (*z* = 1.80, *p* = .07) conditions. Neither model provides a significant fit to the pragmatically solvable conditions.

**Table 10:** Experiment 3. Model evaluation results from the *Listener* task comparing RSA to the baseline literal listener model (LL). All correlations tests exclude predictions for 0 and 100. Models are ranked according to significant fit. cocor-*p*: *p*-value for comparison of overlapping dependent correlations.

Dataset	Listener word	Rank	Model	*r*	$R_{adj}^{2}$	*t*	*p*	cocor-*p*
Overall	color / shape word	–	LL	0.81	0.64	7.69	< .0001	} .73
		–	RSA	0.80	0.62	7.45	< .0001	
	color word	–	RSA	0.77	0.56	4.61	< .001	} .15
		–	LL	0.70	0.46	3.82	< .01	
	shape word	1	LL	0.90	0.80	7.96	< .0001	} < .05
		2	RSA	0.84	0.68	5.88	< .0001	
Pragmatically reducible conds.	color word	–	LL	0.94	0.86	7.14	< .001	} .07
		–	RSA	0.89	0.76	5.15	< .01	
	shape word	–	LL	0.99	0.97	15.72	< .0001	} .07
		–	RSA	0.98	0.95	11.95	< .0001	
Pragmatically solvable conds.	color word	–	RSA	0.06	−0.16	0.14	.89	} < .05
		–	LL	−0.34	−0.04	−0.87	.42	
	shape word	–	LL	0.59	0.24	1.80	.12	} .24
		–	RSA	0.38	0.00	1.01	.35	

When comparing the fit of RSA to a uniform prior version of RSA, we find that in the pragmatically reducible conditions, RSA’s predictions are highly correlated with listener responses (Table 10). By contrast, the fit of the uniform prior version of RSA is almost perfectly anti-correlated with listener responses (color word: *r* = −0.96, $R_{adj}^{2}=0.92$, *t* = −9.64, *p* < .0001; shape word: *r* = −0.99, $R_{adj}^{2}=0.98$, *t* = −21.17, *p* < .0001). These results indicate that the prior helps RSA by completely reversing the pragmatic component’s incorrect predictions. In the pragmatically solvable conditions, the role of the prior differs depending on whether listeners observe a shape or color word. When given a shape word, the fit of the uniform prior version of RSA decreases and reverses direction (*r* = −0.64, $R_{adj}^{2}=0.31$, *t* = −2.04, *p* = .09) relative to RSA. This finding again indicates that the prior helps RSA by overriding the pragmatic component’s incorrect predictions. Conversely, when given a color word, the uniform prior version of RSA (*r* = 0.81, $R_{adj}^{2}=0.59$, *t* = 3.35, *p* < .05) outperforms RSA. This result indicates that the incorporation of the prior in these conditions markedly decreases the fit of RSA’s posterior predictions.

### 6.3 Discussion

In Experiment 3, we set out to increase participant engagement in order to motivate listeners to correctly infer the speaker’s intended referent. To this end, we created a ‘bomb disarming’ scenario in which (fictional) lives depend on making the correct choice. Although participants found the bomb version of the task to be significantly more engaging than the generic version used in Experiment 2, we again only found modest evidence of pragmatic behavior: Listeners responded pragmatically only in the pragmatically solvable conditions, and only when they were given a color word (replicating Experiment 2 and [32]). In the pragmatically reducible conditions, listeners preferred the highly salient (non-pragmatic) competitor both when given a color or a shape word, replicating Experiment 1.

Model evaluation results further revealed an important aspect of the RSA model: RSA does not always predict that listeners will behave ‘pragmatically,’ in the sense that listeners reason about speakers. That is, RSA does not always predict that Listener responses will be proportional to the word’s informativity as defined by the speaker model. The clearest example of this can be seen in the pragmatically reducible conditions, where the predictions of RSA and the baseline literal listener model are quite similar. Critically, this is due to the fact that the prior of the RSA model (as measured by the Salience task) essentially reverses the pragmatic component’s predictions, and thus RSA ultimately predicts that listeners will choose the non-pragmatic referent. Indeed, one may argue that the dominance of the prior in the RSA predictions is motivated by the fact that these conditions examine listener behavior in cases where the speaker is not maximally informative. Importantly, however, we observe that RSA never significantly outperforms the baseline model, which seems to challenge previously established evidence for pragmatic reasoning in one-shot web-based language games.

## 7 General discussion

The investigation of pragmatic reasoning has taken a central role in recent studies of human language processing and in the study of cognitive processes more generally. A highly influential formalization of pragmatic reasoning behavior in the context of referential processing is instantiated by the Rational Speech Act (RSA) model, as proposed by F&G [20]. RSA models human behavior by assuming that a Gricean ‘pragmatic’ listener can use Bayesian inference to resolve a speaker’s intended referent. The *posterior* probability that a given word refers to a particular object in context is based on a combination of (a) the *likelihood* that a speaker would use the word to refer to that object (the ‘pragmatic’ component), and (b) the *prior* probability of referring to each object in the context (as measured by the Salience task). F&G show that despite the highly constrained setting of a one-shot web-based referential communication game, the predictions from the RSA model are strongly correlated with observed human judgments (*r* = 0.99), even when excluding (trivial) predictions of 0 and 100 (*r* = 0.87).

The goal of the current study was to investigate the extent to which the pragmatic reasoning assumed by RSA—i.e., the likelihood as determined by the speaker model—is required to explain human judgments in one-shot web-based communication games. To address this question, we systematically explored a broad range of visual context types to assess which elicit pragmatic behavior in this task. In line with the results reported by F&G, we found a high correlation between Listener responses and RSA’s posterior probabilities. However, when evaluating the performance of RSA relative to a baseline literal listener model that is driven solely by literal word meaning and the prior probability of referring to an object, we found that RSA did not perform better than the baseline model. In fact, we found that listeners only showed evidence of pragmatic reasoning in two out of the 24 conditions (see Fig 3) that were tested across three experiments: namely, when observing a *color* word in the ‘pragmatically solvable’ contexts. These results can be partially explained by the fact that, as in the original study by F&G, a large proportion of the tested conditions utilized context types in which pragmatic reasoning does not aid in resolving the referent because either the literal meaning of the given word uniquely identifies the speaker’s intended referent (the ‘trivial’ conditions in Fig 3), or no amount of pragmatic reasoning can overcome the referential ambiguity in the visual scene (the ‘ambiguous’ conditions in Fig 3). Thus, the added value of RSA’s pragmatic component cannot be evaluated in these conditions, as any model that takes into account literal word meaning—in combination with the prior—will make the same predictions as RSA.

Importantly, focusing on the ‘pragmatic’ conditions, in which the predictions of RSA diverge from those of the baseline literal listener model, reveals two issues regarding the role of pragmatic reasoning in explaining human judgments in referential communication games. First, in the pragmatically solvable conditions, we only find evidence of pragmatic behavior when listeners are presented with a *color* word, but not when they are given a *shape* word. This disparity cannot be explained by RSA (nor by the baseline model). Second, our results indicate that the prior overrules RSA’s pragmatic component in determining the model’s predictions in a large subset of the pragmatic conditions, and as a result RSA is outperformed by the baseline model in these conditions. Below, we discuss these findings in more detail and consider the implications of our results for the study of pragmatic reasoning in language games, and pragmatic communication more generally.

### 7.1 Pragmatically solvable conditions: Shape vs. color

The pragmatically solvable conditions (see Fig 3) were of particular interest in the current study because they serve as the canonical example of pragmatic reasoning in reference games—yet previous work suggests that listeners may not actually employ pragmatic reasoning in such contexts in one-shot web-based experiments [32, 43]. Although we did find evidence of pragmatic reasoning in two out of the four pragmatically solvable conditions (namely, when listeners observed a color word), this evidence was somewhat mixed. In Experiment 1, listeners were at chance in choosing between the pragmatic referent and the color competitor for these conditions, while in Experiments 2 and 3 listeners showed a preference for the pragmatic referent. In addition, the fit of RSA to this subset of the data varied considerably across experiments, from extremely poor and non-significant fits in Experiments 1 and 3, to a reliable correlation of *r* = 0.4 in Experiment 2. For the remaining two solvable conditions (i.e. when listeners were given a shape word), responses showed no evidence of pragmatic reasoning. Instead, listeners preferred the shape competitor over the pragmatic referent, replicating [32].

Given that both word informativity and the prior are constant within the pragmatically solvable conditions, neither RSA nor the baseline literal listener model can explain the difference in Listener behavior between observing a shape versus a color word. Critically, this effect was shown to be consistent across stimuli types (Experiment 2) and under increased participant engagement (Experiment 3). One possible explanation for this effect may have to do with the difference in word class: shapes are generally referred to using nouns (e.g., “fish”), whereas color terms (e.g., “blue”) are typically used as adjectives. Consistent with this generalization, Experiment 1 found that in the Speaker task, participants had a preference for using nouns (shape words) over adjectives (color words) when considering only those contexts where nouns and adjectives were equally informative. This finding replicates [32], who argue that this pattern of speaker behavior arises because it is more natural to use nouns than adjectives when referring to concrete objects with single words.

On this account, listener behavior in these conditions may be influenced by a difference in expectedness between nouns and adjectives. In the shape word conditions, where listeners observe an (expected) noun, their responses pattern with responses from the Salience task. In contrast, in the color word conditions, listeners observe an (unexpected) adjective and this violation of the prior expectation for a noun may trigger additional reasoning that leads participants to select the pragmatic referent. Note that the basic RSA model cannot capture this type of reasoning because the informative speaker model assumed by RSA does not distinguish between word class. In principle, it could be possible to modify RSA to account for differences in speaker preferences. One natural place to make such an adjustment would be in the speaker model itself, which is intended to capture the likelihood that speakers use particular words. However, even if the speaker model was modified to account for such differences in the use of nouns versus adjectives, it would arguably make the inverse prediction: the speaker model would yield a low likelihood for the unexpected adjectives, thereby predicting an increased effect of the prior. An alternative approach for taking speaker preferences into account is offered by [32], who propose adding an additional utterance cost term. However, their Bayesian comparison results comparing the basic RSA model to a variety of extended versions of RSA which include such a cost term suggest that listeners do not take the speaker’s preferences into account. In sum, it is not clear how the basic RSA model could be modified to account for the difference in expectedness of nouns versus adjectives, despite the fact that this seems to be an important factor in explaining pragmatic reasoning behavior in referential communication games.

### 7.2 Role of the prior in RSA

In order to assess the extent to which the correlation between RSA’s predictions and observed listener responses is driven by the prior, the predictions of the RSA model were compared to a version of RSA that assumes a uniform prior. This comparison showed that the prior both hinders and helps RSA, depending on the particular condition. For instance, when listeners are given a color word in pragmatically solvable contexts, the prior decreases the fit of RSA relative to the uniform prior version of RSA. Put differently, the pragmatic component alone provides a better prediction of listener responses than the RSA model as a whole. In contrast, the prior helps RSA when listeners are given a shape word in pragmatically solvable contexts, because the prior overrides the incorrect predictions of RSA’s pragmatic component and leads the model as a whole to accurately predict that listeners will prefer the non-pragmatic referent.

The dominant role of the prior in driving RSA’s listener predictions becomes even clearer when we consider the pragmatically reducible conditions (see Fig 3), in which the given word truth-conditionally matches all three of the objects in the given context, but reasoning about an informative speaker may exclude one of the objects. Arguably, these conditions provide a particularly stringent test of whether listeners reason pragmatically in one-shot reference games, because the reducible conditions effectively pit pragmatic referents against saliency—i.e., the non-pragmatic referent is the only object with a unique feature, and is thus deemed to be highly salient. In other words, listeners essentially decide between making a pragmatic inference or choosing the the object that has the highest prior probability of being referred to. Furthermore, these conditions examine listener behavior in cases where the speaker is not maximally informative. Behavioral results revealed that for both the color and shape word conditions, listeners preferred the salient (non-pragmatic) target. Here again, RSA’s excellent fit to this subset of the data was precisely because the prior reversed the incorrect predictions of the pragmatic component (which were anti-correlated with listener responses) and led RSA to prefer the competitor over both of the pragmatic referents.

Thus, in the majority of the pragmatic conditions we tested in the current study, the prior overrode RSA’s pragmatic component and led RSA to ultimately predict that listeners would prefer the non-pragmatic referent. The importance of the prior is in itself not problematic for RSA. Indeed, the prior is an inherent, mathematically well-motivated, component of rational Bayesian models. However, the findings of F&G have more specifically been taken as compelling evidence that listeners go beyond a simple literal interpretation of the utterance, and reliably reason about rational, informative speakers. In contrast, our findings suggest that the evidence for this conclusion is limited, since overall the RSA model performed no better than the baseline literal listener model.

It is important to note that while the prior, as estimated by the Salience task, includes some aspects of visual salience, it may also implicitly capture a certain degree of reasoning about speakers. That is, in order to obtain a prior for the model comparisons, participants in the Salience task were asked to identify one of the objects in a given visual scene, which was crucially part of a communicative setting in which a speaker tries to communicate one of the objects to the listener. As a result, the prior may incorporate participants’ reasoning about the objects that a speaker is likely to refer to. This means that the predictions from the baseline literal listener model are also driven by a form of reasoning about speaker behavior. Critically, however, this reasoning differs from the type of pragmatic inferencing that RSA aims to formalize using the informative speaker model, which determines the likelihood of using a particular word to refer to an object. It is this latter type of pragmatic reasoning for which we find little evidence in the context of one-shot, web-based referential communication games.

### 7.3 Implications for models of pragmatic reasoning

Taken together, our findings caution against interpreting the high correlation of RSA with human behavior as evidence that listeners reason pragmatically about maximally informative speakers in one-shot web-based referential language games. Our findings instead suggest that a simple literal determination of a word’s likelihood, combined with the prior, offers at least as good (and sometimes better) an estimate of the posterior probability that listeners compute. Although this conclusion is, on the one hand, restricted to the task and setting explored in these one-shot language games, our findings nonetheless highlight the importance of examining the independent contribution of both the likelihood and the prior, in determining the posterior, particularly when theoretical claims are being made for either of those terms individually.

While it might be tempting to suggest that the lack of evidence for pragmatic reasoning is due to the artificial and non-interactive nature of the experimental task and setting, it is important to note that in the Speaker task, participant’s choices did correlate strongly with informativity. This demonstrates that participants—when in the role of speaker—are able to determine the most informative word for a given referent in these settings. Thus, while there is nothing in principle about the setting or stimuli that prevents listeners from modeling informative speakers, it appears that they rarely do so (see also [32], p. 217). In future work, we plan to assess whether methodological factors (e.g., multiple items rather than one, alternating between speaker and listener roles) modulate the prevalence of pragmatic processing in this task.

As we have noted, our literal listener model simply replaces the informative speaker model assumed by RSA while staying within a broader Bayesian formulation of the problem. First and foremost, we proposed the literal listener model as a baseline against which we could better evaluate and understand the contribution of the informative speaker model—and thus pragmatic inference—to RSA’s performance. Given the strong performance of this baseline, however, it is worth discussing its plausibility as a cognitive model. Specifically, the literal listener model can be viewed as a bounded-rational listener who resolves the intended referent based solely on the literal content of the utterance and the prior. One way to reconcile this model with RSA would be to suggest that RSA is best viewed as a *computational* level theory [55], reflecting what an ideal listener should compute in order to optimize their understanding of a rational speaker. Computational level theories typically emphasize what must be computed, and why, with the latter being determined by the goal of optimizing success in the task at hand. The aim of such a rational approach is to identify how people should ideally behave, while typically acknowledging that bounds on cognitive resources may result in people only approximating the computational theory. This more mechanistic account of how people actually accomplish the task is referred to as the *algorithmic* level theory. It might be reasonable, therefore, to suggest that the literal listener model corresponds to such an algorithmic account, in which the likelihood function is approximated using the simple literal meaning, eschewing the full reasoning about various contextual factors that would have led the speaker to use more informative encodings. Such a perspective is particularly plausible to the extent that the evidence for pragmatic reasoning about speaker behavior in other domains or tasks is more clear than that found in the current study. It then remains simply to be identified what factors (e.g., engagement, complexity, resource bounds, etc.) determine the degree of rationality listeners realize. Parameterisations of RSA’s speaker rationality parameter *α* and depth of recursion (e.g., whether speakers model listeners that reason about rational speakers, etc.) can be see seen as characterizing degrees of rational communication, with the simplest of these being our literal listener.

## 8 Conclusion

Referential communication games, in which speakers aim to communicate objects from a given referential context to listeners, are widely used experimental designs for investigating pragmatic communication in a controlled setting. Frank & Goodman [20] showed that the predictions that derive from the RSA model—which combines pragmatic reasoning about an informative speaker with a prior— strongly correlate with human judgments in a web-based one-shot version of the referential communication game. This result has been taken as compelling evidence that listeners reason pragmatically about an informative speaker. The current set of studies shows that the high correlation of RSA with human behavior can be explained by factors other than the pragmatic reasoning implemented by RSA: when comparing RSA to a baseline literal listener model that is driven solely by literal word meaning and a prior estimating contextual salience, we find that the baseline model largely outperforms RSA. This finding is due to the fact that (a) in a large number of the conditions used by Frank & Goodman (and our Experiment 1), the literal meaning of a word uniquely identifies the referent, and (b) when it does not, the better predictor for listener behavior seems to be the salience prior, which informs both RSA and the baseline model, rather than the speaker model formalized by RSA. Indeed RSA often accurately predicts that listeners will behave non-pragmatically, precisely because the prior overrides the speaker model. While the constrained nature of one-shot web-based language games may contribute to this behavior in participants playing the role of the Listener, participants playing the role of the Speaker do behave rationally in determining the most informative word for a given referent in this setting. This leads us to conclude that RSA’s strong overall correlation with human behaviour should not be taken as evidence that listeners reliably reason about the intentions of informative speakers in these settings. While richer communicative settings may elicit more systematic pragmatic behavior in language games, our findings nonetheless highlight the importance of examining the independent contribution of the individual model components in describing listener behavior, particularly when theoretical claims are being made for either of those terms individually.

## Supporting information

S1 Data Data from Experiment 1. (CSV)

S2 Data Data from Experiment 2. (CSV)

S3 Data Data from Experiment 3. (CSV)

S1 File (PDF)

S2 File (ZIP)
