# Supplementary material for: Reevaluating pragmatic reasoning in language games
Source: PLoS One. 2021 Mar 17;16(3):e0248388. doi: 10.1371/journal.pone.0248388 (PMC7968720; doi:10.1371/journal.pone.0248388)
Supplement: S1 File — (PDF) [file pone.0248388.s004.pdf]

S1 File

S1 Experimental Procedure. The current set of experiments was implemented via a custom script and hosted on a local server at Saarland University. Following informed consent participants were randomly assigned to either the Speaker (not included in Experiments 2 and 3), Listener, or Salience task, which opened within a new browser window. Participants then proceeded through a series of self-paced screens using a button labeled “Next” (see Fig 6). In Experiments 1 and 2, the first screen presented participants with a cartoon picture of a man and text that read, “This is Robert. You will be interacting with him in this HIT.” In the Experiment 3, the introduction consisted of two screens: the first screen showed a picture of a cartoon villain and a bomb, and a text that read “The city you live in is in great danger! The super villain Dr. Sinisterioso has planted a bomb and it is about to go off!”. The second screen showed a cartoon character wearing a bomb suit, together with the following text: “This is Lt. Robertson. He’s on the bomb squad. He just found Dr. Sinisterioso’s secret bomb manual, but only you have access to the bomb itself. You have to work together with Lt. Robertson to disarm it in time! You can only talk to each other via text message.”

Next, an attention check was performed, which was designed to test whether participants were paying attention to the task and to minimize the possibility that a bot could pose as a worker. An item (e.g., green boot, green fish, blue fish) was randomly selected from one of the 24 visual context types described in Section 3 and was presented on the screen. In Experiment 3, the objects were presented as buttons on a cartoon bomb that had a (non-counting) timer, together with the text “To make sure Lt. Robertson knows what type of bomb it is, you need to tell him the button configuration!”/ Two attention check questions appeared along with this visual context (Exp. 1 & 2 : e.g., “How many objects are fish/blue?”; Exp. 3: e.g., “How many buttons have fish/blue symbols?”). To create these questions, a feature (e.g., shape) was randomly selected from the target object (e.g., green fish). Then one of the competitor objects was randomly selected (e.g., blue fish) and the other feature (e.g.,

color) was selected for second question. Thus, in order to answer the attention questions correctly, participants had to attend to the colors and shapes of both the target and its competitors. The order of questions was counterbalanced such that for each visual context type, the shape question was listed first for half of the participants and the color question was listed first for the other half. This also ensured that the questions did not give away which object was the target. Participants responded to each question by selecting from four possible values (0, 1, 2, 3) using a drop-down menu. Participants who answered either question incorrectly were excluded from analyses. The "Next" button was enabled only after participants responded to both questions. This was indicated by changing the color of the button from gray to blue.

The screen containing the critical task presented the same visual context as the attention screen, with each object designated by a letter (A, B, C). In Experiment 3, only the buttons containing the objects were shown and not the bomb itself. The text varied depending on which task the participant had been assigned to. Continuing with the same example item as above, in the Speaker task (Experiment 1) participants read: "Imagine you are talking to Robert and you want him to pick out Item B. If you can only use one word, which word would you say?". Participants then made a forced choice between the color word (green) and shape word (fish) by clicking a radio button. The order of color vs. shape options was counterbalanced across trials. In the Listener task (Experiment 1 & 2) participants read: "Robert wants you to pick one of the objects above but he can only say one word. He says: [fish/green]. Which object do you think he is talking about?". The word given by Robert was counterbalanced such that half of the participants saw a shape word and half saw a color word. Participants then made their choice by clicking one of three radio buttons labeled A, B, and C. In Experiment 3, only the formulation of the task differed: "Time is running out so Lt. Robertson can only send a single word to disarm the bomb ... [fish/green]. Which button do you press?". Finally, in the Salience task participants read: "Robert wants you to pick one of the objects above, but due to background noise you cannot understand what he said. Which object do you think he is most likely talking about?" (Experiment 1 & 2), or "Time is running out so Lt. Robertson can only send a single word to disarm the bomb ... Oh no! Dr. Sinisterioso has jammed communications so you can't read his text! Which button do you press?" (Experiment 3). Participants made their choice by clicking one of three radio buttons labeled A, B, and C. In each task, the "Next" button was only enabled once participants made their selection.

The final screen was a brief exit survey, which contained the questions shown in

S1 Table. Exit survey questions for Experiment 1-3

Experiment	Question	Question Type
1,2	What was the name of the character you interacted with in this HIT?	drop-down
3	What was the name of your bomb squad partner?	drop-down
1,2	Which objects did you see? (select as many as apply)	radio buttons
3	Which symbols did you see on the buttons? (select as many as apply)	radio buttons
1-3	Please tell us about yourself. *You will not be penalized for your answers.*	
1-3	Age range	drop-down
1-3	Gender	drop-down
1-3	Native language	drop-down
1-3	Fluency in English	drop-down
2,3	How engaging did you find this HIT?	slider (*not at all – very much*)
1-3	What do you think this HIT was about?	text field
1-3	Any other comments for us?	text field

S1 Table. Participants who did not select “English” for the native Language question or “Fluent” for the fluency in English question were excluded from analyses. Upon completing the HIT, participants were given a randomly generated five digit alpha-numeric code to paste back into the Mechanical Turk interface in order to be compensated.
