# Supplementary figures and images for: Reevaluating pragmatic reasoning in language games
Source: PLoS One. 2021 Mar 17;16(3):e0248388. doi: 10.1371/journal.pone.0248388 (PMC7968720; doi:10.1371/journal.pone.0248388)

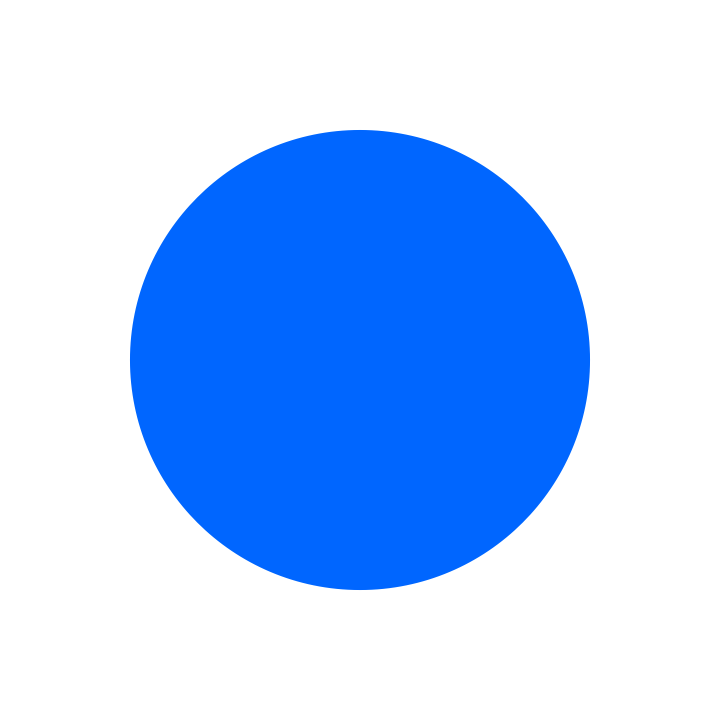

Supplement: S2 File — (ZIP) [file pone.0248388.s005.zip › images/blue.circle.png]

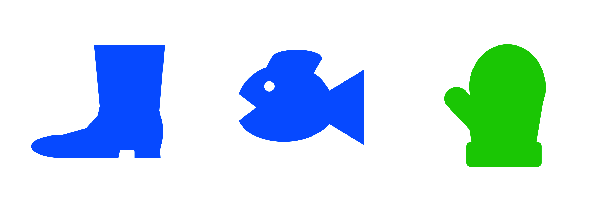

Supplement: S2 File — (ZIP) [file pone.0248388.s005.zip › images/1s2c.ds.dc.png]

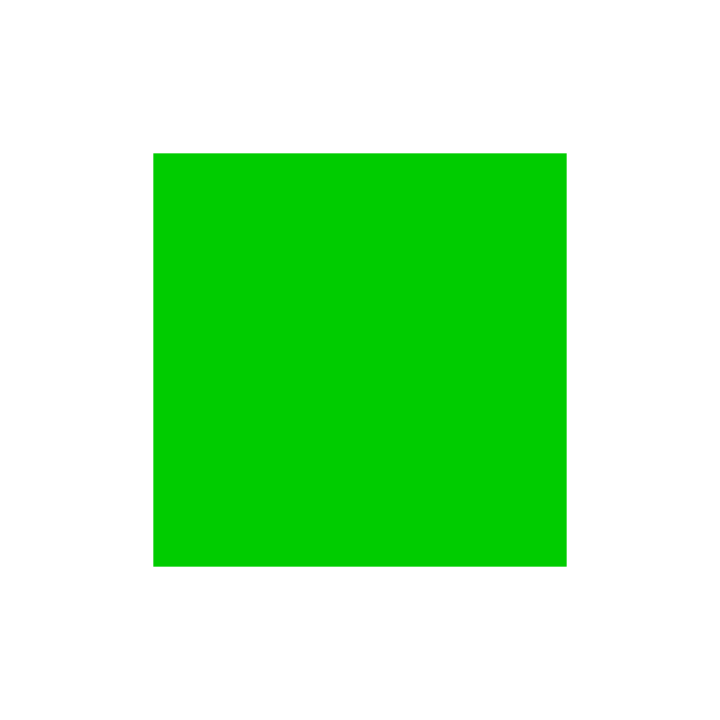

Supplement: S2 File — (ZIP) [file pone.0248388.s005.zip › images/green.square.png]

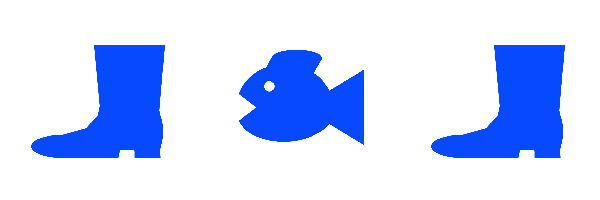

Supplement: S2 File — (ZIP) [file pone.0248388.s005.zip › images/1s3c.ss.sc.png]

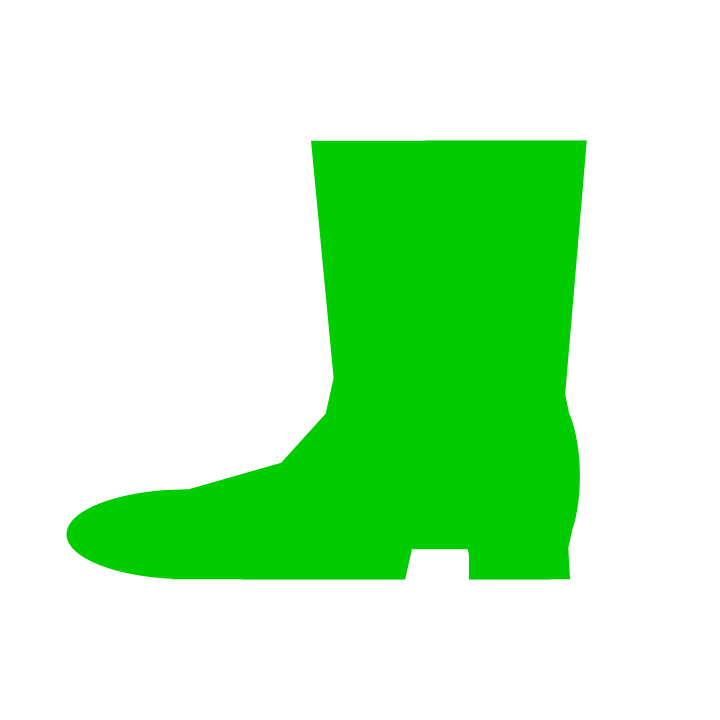

Supplement: S2 File — (ZIP) [file pone.0248388.s005.zip › images/green.boot.png]

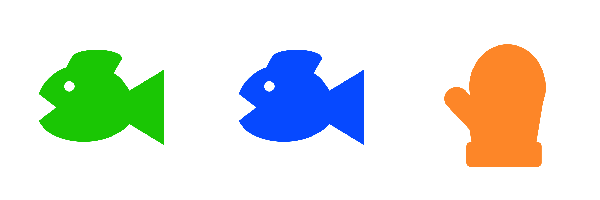

Supplement: S2 File — (ZIP) [file pone.0248388.s005.zip › images/2s1c.ds.dc.png]

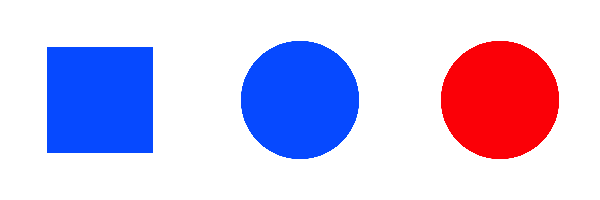

Supplement: S2 File — (ZIP) [file pone.0248388.s005.zip › images/FG_12.png]

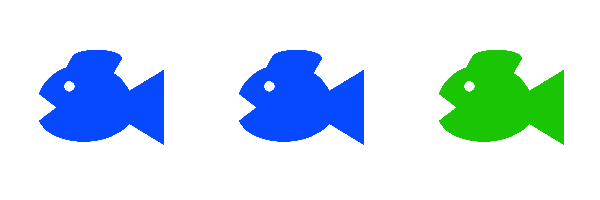

Supplement: S2 File — (ZIP) [file pone.0248388.s005.zip › images/3s2c.png]

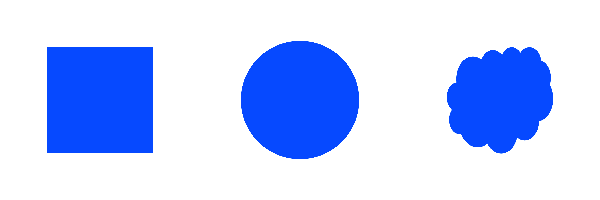

Supplement: S2 File — (ZIP) [file pone.0248388.s005.zip › images/FG_13.png]

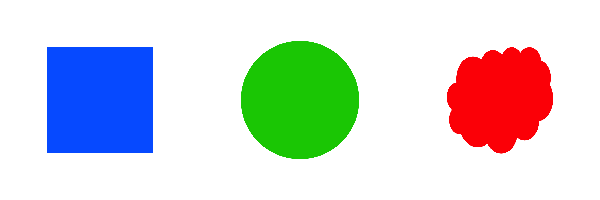

Supplement: S2 File — (ZIP) [file pone.0248388.s005.zip › images/FG_11.png]

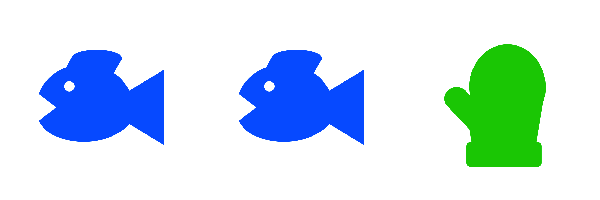

Supplement: S2 File — (ZIP) [file pone.0248388.s005.zip › images/2s2c.a.png]

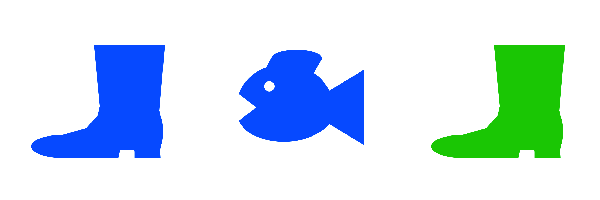

Supplement: S2 File — (ZIP) [file pone.0248388.s005.zip › images/1s2c.ss.dc.png]

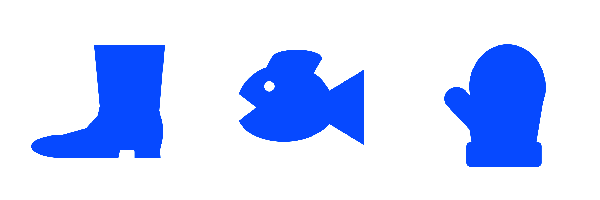

Supplement: S2 File — (ZIP) [file pone.0248388.s005.zip › images/1s3c.ds.sc.png]

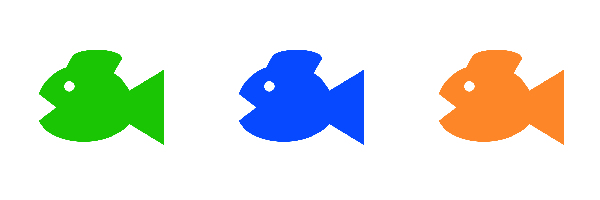

Supplement: S2 File — (ZIP) [file pone.0248388.s005.zip › images/3s1c.ss.dc.png]

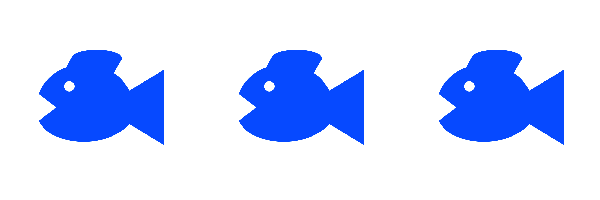

Supplement: S2 File — (ZIP) [file pone.0248388.s005.zip › images/3s3c.png]

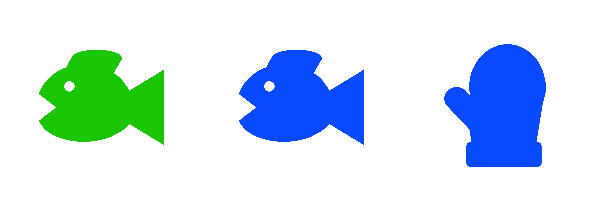

Supplement: S2 File — (ZIP) [file pone.0248388.s005.zip › images/2s2c.b.png]

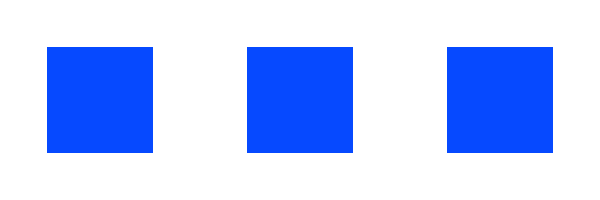

Supplement: S2 File — (ZIP) [file pone.0248388.s005.zip › images/FG_33.png]

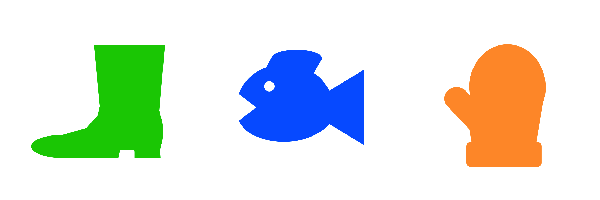

Supplement: S2 File — (ZIP) [file pone.0248388.s005.zip › images/1s1c.png]

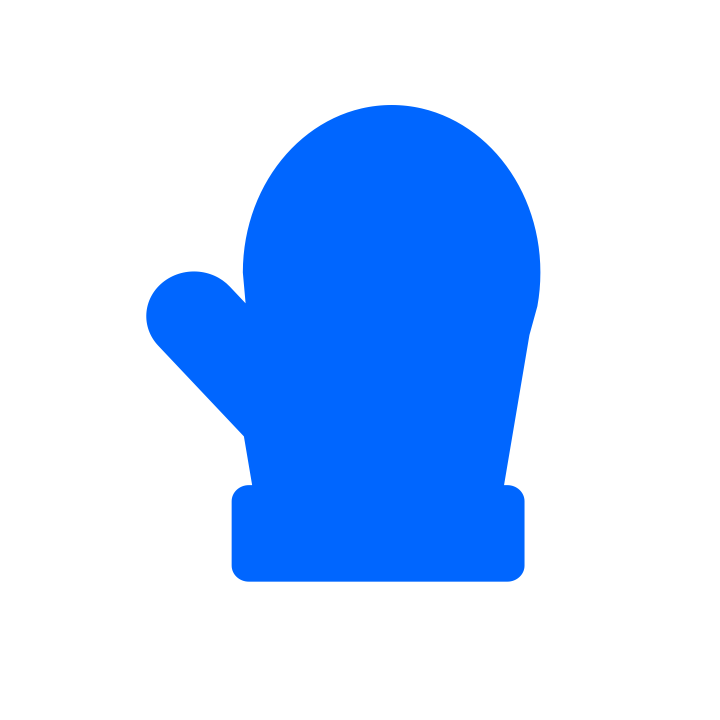

Supplement: S2 File — (ZIP) [file pone.0248388.s005.zip › images/blue.mitt.png]

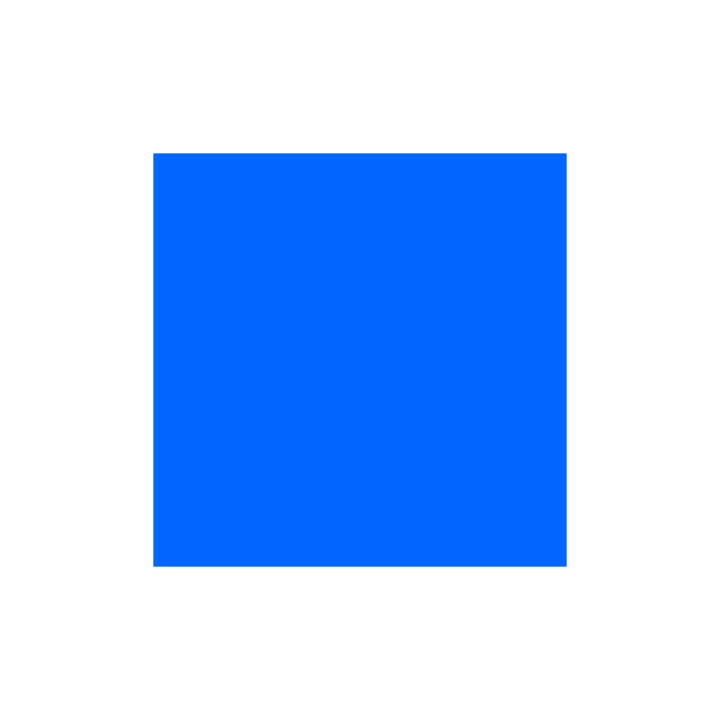

Supplement: S2 File — (ZIP) [file pone.0248388.s005.zip › images/blue.square.png]

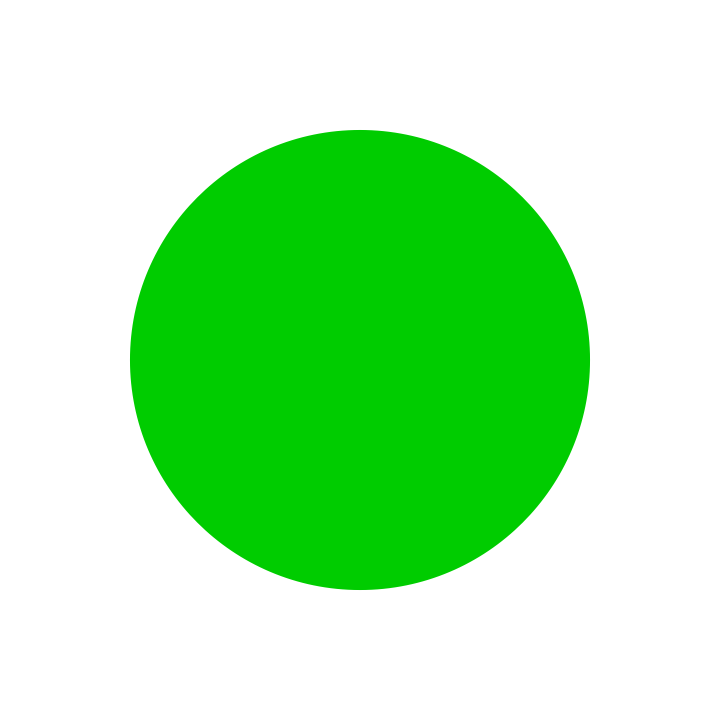

Supplement: S2 File — (ZIP) [file pone.0248388.s005.zip › images/green.circle.png]

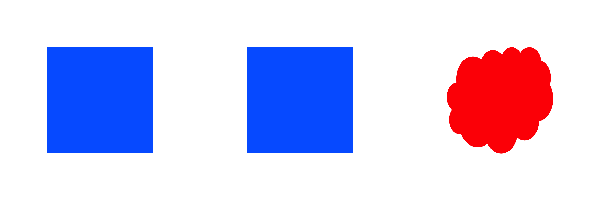

Supplement: S2 File — (ZIP) [file pone.0248388.s005.zip › images/FG_22a.png]

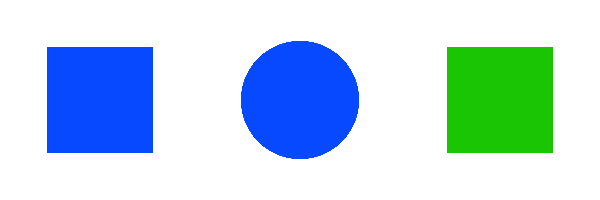

Supplement: S2 File — (ZIP) [file pone.0248388.s005.zip › images/FG_22b.png]

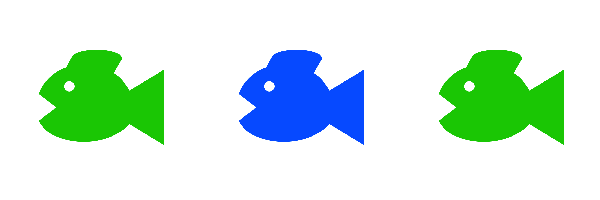

Supplement: S2 File — (ZIP) [file pone.0248388.s005.zip › images/3s1c.ss.sc.png]

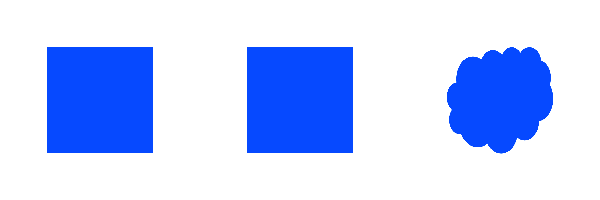

Supplement: S2 File — (ZIP) [file pone.0248388.s005.zip › images/FG_23.png]

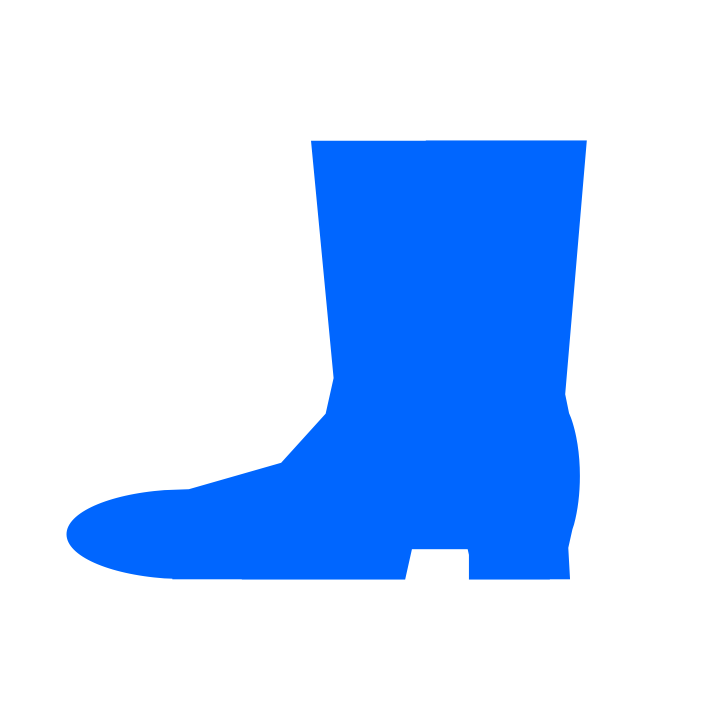

Supplement: S2 File — (ZIP) [file pone.0248388.s005.zip › images/blue.boot.png]

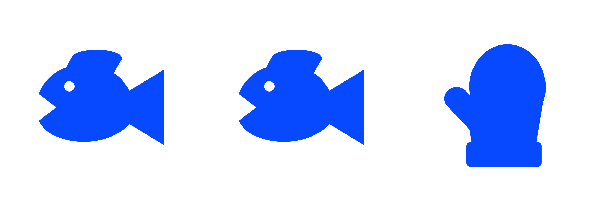

Supplement: S2 File — (ZIP) [file pone.0248388.s005.zip › images/2s3c.png]

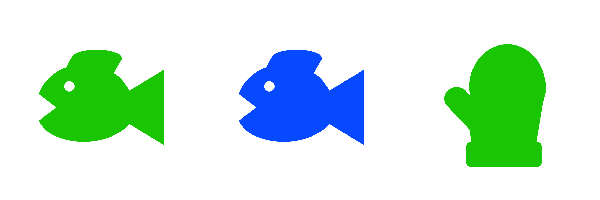

Supplement: S2 File — (ZIP) [file pone.0248388.s005.zip › images/2s1c.ds.sc.png]
